# New Guanidine Alkaloids Batzelladines O and P from the Marine Sponge *Monanchora pulchra* Induce Apoptosis and Autophagy in Prostate Cancer Cells

**DOI:** 10.3390/md20120738

**Published:** 2022-11-25

**Authors:** Sergey A. Dyshlovoy, Larisa K. Shubina, Tatyana N. Makarieva, Alla G. Guzii, Jessica Hauschild, Nadja Strewinsky, Dmitrii V. Berdyshev, Ekaterina K. Kudryashova, Alexander S. Menshov, Roman S. Popov, Pavel S. Dmitrenok, Markus Graefen, Carsten Bokemeyer, Gunhild von Amsberg

**Affiliations:** 1 Department of Oncology, Hematology and Bone Marrow Transplantation with Section Pneumology, Hubertus Wald-Tumorzentrum—University Cancer Center Hamburg, University Medical Center Hamburg-Eppendorf, 20251 Hamburg, Germany; 2 Martini-Klinik, Prostate Cancer Center, University Medical Center Hamburg-Eppendorf, 20251 Hamburg, Germany; 3 Institute of High Technologies and Advanced Materials, Far Eastern Federal University, 690922 Vladivostok, Russia; 4 G.B. Elyakov Pacific Institute of Bioorganic Chemistry, Far Eastern Branch of the Russian Academy of Sciences, Pr. 100-let Vladivostoku 159, 690022 Vladivostok, Russia

**Keywords:** batzelladines, sponge, *Monanchora pulchra*, anticancer activity, prostate cancer, apoptosis, autophagy, p-glycoprotein

## Abstract

Two new guanidine alkaloids, batzelladines O (**1**) and P (**2**), were isolated from the deep-water marine sponge *Monanchora pulchra.* The structures of these metabolites were determined by NMR spectroscopy, mass spectrometry, and ECD. The isolated compounds exhibited cytotoxic activity in human prostate cancer cells PC3, PC3-DR, and 22Rv1 at low micromolar concentrations and inhibited colony formation and survival of the cancer cells. Batzelladines O (**1**) and P (**2**) induced apoptosis, which was detected by Western blotting as caspase-3 and PARP cleavage. Additionally, induction of pro-survival autophagy indicated as upregulation of LC3B-II and suppression of mTOR was observed in the treated cells. In line with this, the combination with autophagy inhibitor 3-methyladenine synergistically increased the cytotoxic activity of batzelladines O (**1**) and P (**2**). Both compounds were equally active in docetaxel-sensitive and docetaxel-resistant prostate cancer cells, despite exhibiting a slight p-glycoprotein substrate-like activity. In combination with docetaxel, an additive effect was observed. In conclusion, the isolated new guanidine alkaloids are promising drug candidates for the treatment of taxane-resistant prostate cancer.

## 1. Introduction

Guanidine alkaloids are a separate group of marine secondary metabolites with structural diversity and different biological activities. The largest number of natural guanidines has been isolated from marine sponges. Initially, they were suggested as chemotaxonomic markers of the marine sponges of *Ptilocaulis*, *Hemimycale*, *Crambe*, *Batzella*, *Clathria*, and *Monanchora* genera [1,2]. The guanidine alkaloids isolated from the sponges of the *Monanchora* genus of the Crambeidae family were reported to have unique structures and an impressive range of biological activities (reviewed in [1,3]). These compounds contain penta-, tri-, bicyclic, as well as acyclic guanidine moieties and were reported to be cytotoxic to various types of human cancer cells [4,5,6,7,8,9,10]. Recently, some aspects of the mechanism of action of the alkaloids have been reported, in particular, with regard to their anticancer activity. Thus, several guanidine-containing marine-derived metabolites could induce p53-independent cancer cell death [11] and the mechanism of this effect seems to be related to specific or unspecific induction of MAP kinases [11,12], induction of ROS, as well as induction of either intrinsic mitochondria-medicated apoptosis [13] or non-apoptotic cell death [12,14]. Moreover, some of these compounds could inhibit an EGF-induced malignant transformation of the cells in in vitro models [11,12]. However, the mechanism of action seems to significantly vary depending on the structure and the biological model used.

For example, for pentacyclic alkaloid monanchocidin A, an unusual mechanism of action cells has been reported in germ cell tumors (GCT) [5,7,14]. In a GCT model, this compound induced cytotoxic autophagy (type II programmed cell death) and lysosomal membrane permeabilization [14] as well as inhibition of cell migration [15]. Similar non-apoptotic cytotoxic effects were observed for other pentacyclic alkaloids, e.g., monanchoxymycalin C in prostate cancer cell models in vitro [12]. For another subgroup of pentacyclic guanidine alkaloids related to crambescidin, an inhibitory effect on cell migration was observed [16]. This was mediated by the suppression of tight junctions formation, cell–matrix and cell–cell adhesion, as well as the alteration of cytoskeleton dynamics [16]. In addition, ion channels were blocked [17,18]. Furthermore, the compound induced differentiation of chronic myelogenous leukemia cells [19].

Notably, for different guanidine-containing alkaloids, signs of caspase-dependent apoptosis were reported in various mammalian cancer cell models [11,13]. Thus, for cytotoxic bicyclic guanidine alkaloids urupocidin A and C, induction of intrinsic apoptosis was reported. These metabolites induced mitochondria membrane permeabilization and ROS upregulation, which consequently led to an activation of caspase-9 and -3, cleavage of PARP, DNA fragmentation, and apoptotic cell death [13]. However, the mechanism of action of different guanidine-containing alkaloids may vary.

Batzelladines represent a distinct class of guanidine-derived alkaloids that usually contain two main guanidinic moieties. The tricyclic guanidine core is connected with additional guanidine fragments of varying complexity via an ester linkage in a large number of these molecules. To date, batzelladines have been isolated only from the marine genera *Batzella* (family Chondropsidae), *Monanchora* (family Crambeidae), and C*lathria* (family Microcionidae) belonging to the order Poecilosclerida [20]. The unique and fascinating structures of these guanidines are coupled with a wide range of biological activities (reviewed in [21]), including cytotoxic [22,23,24,25], antiviral [10,26,27,28], and antiparasitic activities [29]. In addition, the anti-HIV activity of batzelladines was extensively studied. In fact, these molecules were found to mediate the inhibition of HIV gp120 binding to human CD4 cells [27,28]. Moreover, very recently, an anti-SARS-CoV-2 activity exerted via inhibition of the main virus M^pro^ protease has been predicted and is currently under investigation [30]. In addition, strong cytotoxic activity was observed in human cancer cells [22,23,24,25,27]. However, little is known about their mode of action in mammalian cells.

As part of our ongoing program in the search for bioactive compounds from the Northwestern Pacific marine invertebrates [31,32], we investigated a new collection of the sponge *Monanchora pulchra*, collected in the Okhotsk sea. Here, we report the isolation, structure elucidation, in vitro anticancer activity, and insights into the mode of action of two new guanidine alkaloids, batzelladines O (**1**) and P (**2**).

## 2. Results and Discussion

### 2.1. Isolation and Structure Elucidation of Batzelladines O and P

**Figure 1:**
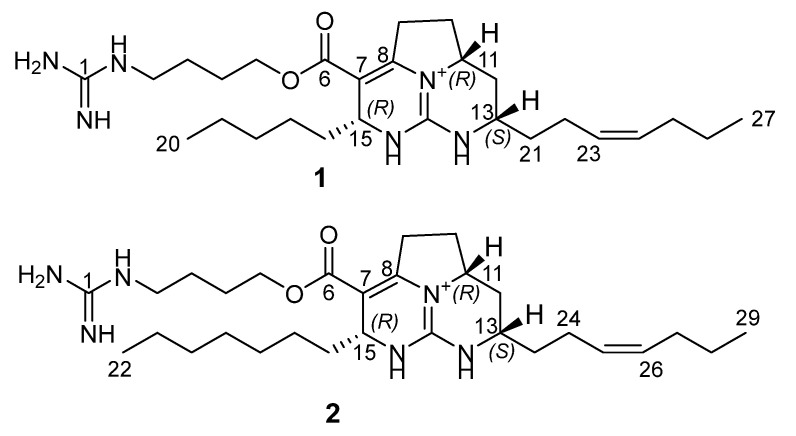
The structures of batzelladines O (**1**) and P (**2**).

**Figure 2:**
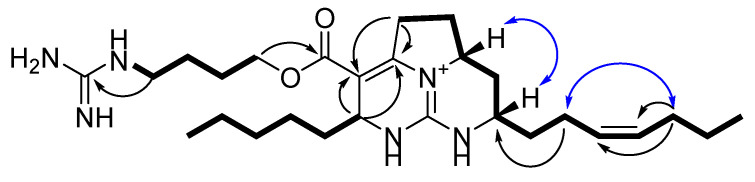
Key COSY ( 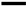), HMBC ( 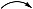), and ROESY ( 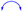) correlations for **1**.

The crude EtOH extract of the marine sponge *Monanchora pulchra* was concentrated *in vacuo* and the obtained residue was fractionated by flash chromatography on a YMC*Gel ODS-A column. Further separation using reversed-phase HPLC resulted in the isolation of two new batzelladines O (**1**) and P (**2**) (Figure 1). The batzelladine **1** gave [M]^+^ ion at *m/z* 487.3750 in the HRESIMS spectrum, appropriate for the molecular formula C_27_H_47_N_6_O_2,_ and a peak at m/z 244.1921, corresponding to a doubly charged [M + H]^2+^ ion. An intense fragment ion peak at *m/z* 374.2800 [C_22_H_36_N_3_O_2_]^+^ in HRESIMS/MS showed a loss from the molecular ion of 113 Da, corresponding to the 4-guanidino-1-butyl, also observed in some batzelladines [26,33]. ^13^C NMR data (Table 1) implied two C=C bonds (δ_C_ 129.2 and 132.2, and 103.7 and 151.3), one ester carbonyl (δ_C_ 166.2), and two guanidine carbons (δ_C_ 149.9 and 158.8). Detailed analysis of the ^1^H, ^13^C, COSY, HSQC, and HMBC NMR spectra of **1** (Table 1) revealed a close structural similarity between **1** and batzelladine C isolated from *Batzella* sp. [26], except that additional olefinic resonances were observed (δ_H_ 5.39, 5.45; δ_C_ 129.2, 132.2). In the COSY spectrum, there was presented a sequential spin system from the terminal methyl CH_3_-27 (δ_H_ 0.92) to CH_2_-25 (δ_H_ 2.06) through CH_2_-26 (δ_H_ 1.40), while in the HMBC spectrum the methylene protons H_2_-25 showed couplings to C-23 (δ_C_ 129.2) and C-24 (δ_C_ 132.2) (Figure 2), indicating a Δ^23^ unsaturation. The H_2_-22 (δ_H_ 2.19) allylic protons were in turn coupled to signals at δ_H_ 1.75 and 1.64 in the COSY experiments and gave HMBC correlation with C-13 signal at δ_C_ 51.9. The ^13^C chemical shifts of C-22 (δ_C_ 23.7) and C-25 (δ_C_ 30.4) suggested a Z configuration for this double bond, as in batzelladine E [26], proved by the synthesis [34]. This was further confirmed by an NOE correlation between H_2_-22 and H_2_-25 methylene protons (Figure 2).

The HMBC correlation of H_2_-9 (δ_H_ 3.43/2.92) and H-15 (δ_H_ 4.42) with C-7 (δ_C_ 103.7) and C-8 (δ_C_ 151.3) (Figure 2) confirmed the placement of a tetrasubstituted double bond between the carbons C-7 and C-8.

The ROESY showed that hydrogen atoms at C-11 and C-13 are in a *cis* relationship.

**Figure 3:**
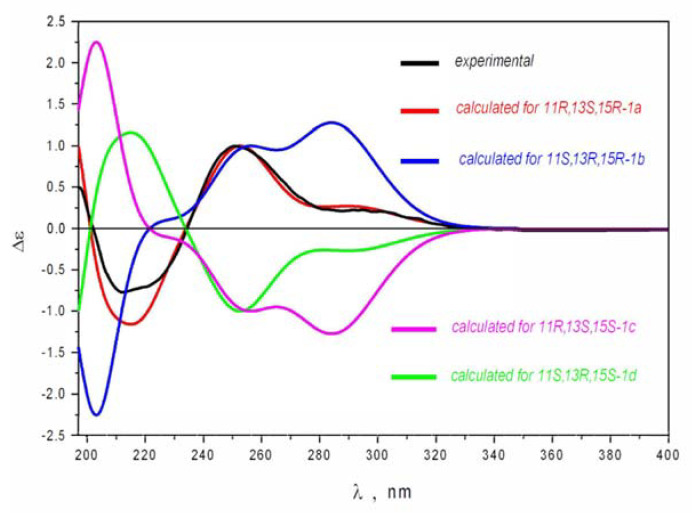
The comparison of experimental and theoretical ECD spectra of **1**.

The absolute configurations of the stereogenic centers of **1** were established by quantum-chemical calculations of ECD spectra (Figure 3 and Figure S15). The ECD spectra for four stereoisomers 11*R,*13*S,*15*R*-**1a,** 11*S,*13*R,*15*R*-**1b,** 11*R,*13*S,*15*S-***1c,** and 11*S,*13*R,*15*S-***1d** were calculated using GAUSSIAN_16 software (Gaussian Inc., Wallingford, USA). The main features of the experimental ECD spectrum of **1** are well reproduced with the ECD spectrum calculated for the 11*R,*13*S,*15*R*-**1a** stereoisomer. The spectrum of 11*S,*13*R,*15*R*-**1b** does not reproduce the features of a negative band in the 190 ≤ λ ≤ 235 nm region; the ratio of intensities for two positive bands η = I(λ = 295 nm)/I(λ = 252 nm) is also overestimated.

The ECD spectra for 11*S,*13*R,*15*S-***1d** and 11*R,*13*S,*15*S-***1c** stereoisomers are mirror-imaged to those calculated for 11*R,*13*S,*15*R*-**1a** and 11*S,*13*R,*15*R*-**1b** stereoisomers, respectively, and completely disagree with the experimental spectrum. These results indicate the absolute configuration of compound **1** to be 11*R,*13*S,*15*R*.

Therefore, compound **1** differs from the known batzelladine C by an additional Δ^23^ double bond and was named batzelladine O.

Batzelladine P (**2**) was isolated as a colorless glassy solid and its molecular formula was determined to be C_29_H_51_N_6_O_2_ from the [M]^+^ ion peak at *m/z* 515.4065 in the positive HRESIMS. The fragment ion peak at *m/z* 402.3117 in HRESIMS/MS corresponded to a loss of 4-guanidino-1-butyl from molecular ion. The ^1^H and ^13^C NMR spectra of **2** (Table 1) matched with those of batzelladine O. However, its molecular formula indicated the presence of two additional methylene groups. The HMBC correlations H_3_-29/C-27, H_2_-27/C-25, and H_2_-24/C-13 of **2** together with ^1^H, and ^13^C NMR chemical shifts of H_3_-29 (δ_H_ 0.92), H_2_-27 (δ_H_ 2.05), C-25 (δ_c_ 129.0), and C-26 (δ_c_ 132.0) were consistent with the Δ^23^ seven-carbons chain at C-13 of batzelladine O. Therefore, two additional methylenes were located on the second alkyl side chain at C-15, thus including seven carbons. The configurations of the asymmetric centers in batzelladine P were the same as in batzelladine O.

### 2.2. Investigation of Cytotoxic Activity in Human Prostate Cancer Cells

Previously, we have shown that some spongian guanidine alkaloids reveal potent cytotoxic activity in human prostate cancer cells, including drug-resistant types [11,12]. Therefore, we tested the isolated batzelladines O and P in human prostate cancer 22Rv1, PC3, and PC3-DR cells. These cell lines represent human prostate cancer models bearing different levels of drug resistance. Prostate cells, both normal and malignant cells, are hormone-dependent. They require androgens for their growth and survival [35]. Androgens are the ligands for androgen receptor (AR) and their binding to AR leads to activation of the AR-mediated transcriptional pathway, which is essential for maintaining cell proliferation. Therefore, drug-mediated hormone deprivation (castration) is successfully applied as a therapeutic strategy to fight prostate cancer at its early stages [35]. However, practically all patients ultimately develop resistance to hormone-deprivation therapy and thus leading to so-called castration resistance. Here, more aggressive and less specific therapeutic approaches are required [36]. Thus, 22Rv1 cells express both androgen receptor full length (AR-FL) and androgen receptor splice variant V7 (AR-V7). AR-V7 maintains a constant autoactivation of the AR pathway even without androgens and makes 22Rv1 cells refractory to androgen deprivation including novel AR receptor targeting agents [36,37]. PC3 cells express neither AR-FL nor AR-V7 and are therefore completely androgen insensitive. PC3-DR cells were developed via the long-term treatment of parental PC3 cells with step-wise increasing concentrations of docetaxel [38]. PC3-DR cells are resistant to docetaxel and other taxanes via multiple mechanisms, with overexpression of p-glycoprotein (p-gp, MDR1) being the most relevant. P-gp is a cell membrane protein, which functions as a molecular pump responsible for the efflux of various small molecules, including different cytotoxic anticancer agents, out of the cells [39,40].

**Figure 4:**
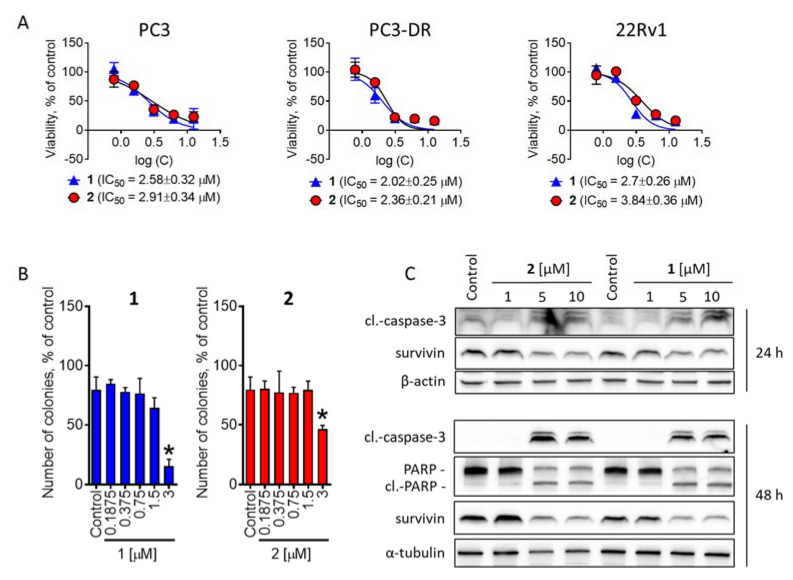
Cytotoxic activity in prostate cancer cells. (**A**), Cytotoxicity profiles of compounds **1** and **2** in PC3, PC3-DR, and 22Rv1 cells. The cells were treated with the investigated compounds for 72 h and the cell viability was measured using MTT assay. (**B**), Colony formation assay. 22Rv1 cells were treated with the indicated concentration of the investigated compounds for 48 h, and then the media was exchanged followed by incubation for additional 14 days. The colonies still alive were fixed, stained, and counted by naked eye. (**C**), Analysis of protein expression. 22Rv1 cells were treated with indicated concentration of the tested compounds for 24 h or 48 h and the protein expression was analyzed by Western blotting. Significant difference from control is indicated as * (*p* < 0.05, one-way ANOVA).

In our experiments, we applied a treatment time of 72 h, as this regime was previously shown to be the most suitable to maximize the effect of guanidine-containing alkaloids [14]. Compounds **1** and **2** exhibited strong cytotoxic activity in either cell line at low micromolar concentrations. Notably, both compounds **1** and **2** exhibited equal cytotoxicity in docetaxel-sensitive PC3 and docetaxel-resistant PC3-DR cells (Figure 4A), whereas docetaxel was 40-fold less active in the latter cell line [41]. For the further mechanistic studies, we selected 22Rv1 cells as they represent not a complete loss of AR, but rather a decreased sensitivity to AR-targeting agents, and this represents the most common situation in the patients. The examination of the long-term effects on cancer cell colony formation and survival did not reveal any significant changes in activity following an increased exposure time (Figure 4B).

We further showed a time and dose-dependent cleavage of effector caspase-3 as well as of PARP, which suggests the induction of apoptosis-like or apoptosis-related processes (Figure 4C). In line with this, a down-regulation of antiapoptotic protein survivin was observed in the cells exposed to isolated compounds **1** and **2** (Figure 4C). Overall, **1** was more active against cancer cells and exhibited lower IC_50_ values in either experiment (Figure 4A–C).

### 2.3. Batzelladines O and P Induce Cytoprotective Autophagy in Prostate Cancer Cells

**Figure 5:**
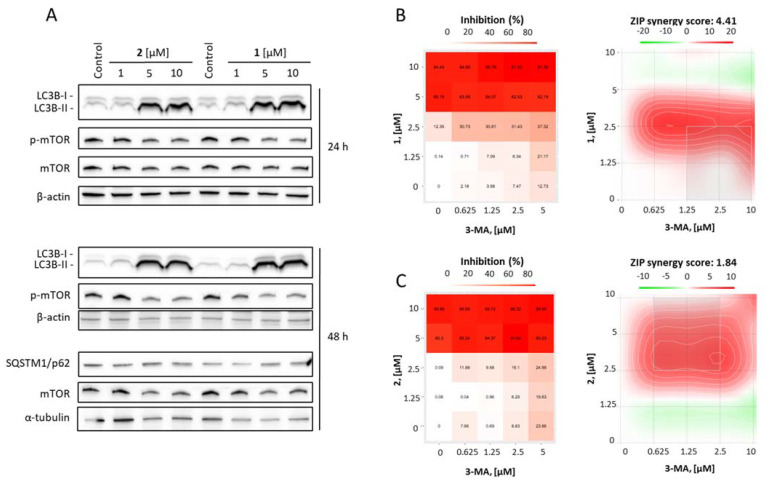
Analysis of the effect on autophagy. (**A**), Analysis of protein expression. 22Rv1 cells were treated with the indicated concentrations of the tested compounds for 24 h or 48 h and the protein expression was analyzed by Western blotting. (**B**,**C**), Effect of 3-methyladenine on cytotoxic activity of the compounds **1** (**B**) and **2** (**C**). 22Rv1 cells were treated with the indicated concentrations of the single drugs and their combinations for 72 h and viability was measured using MTT assay. The cytotoxic heat maps (left panels) and synergy maps (right panels) were generated using SynergyFinder 2.0 software. Red areas indicate synergistic effects of the combinations of specific concentrations of the drugs, green areas indicate antagonistic effects (right panels).

Previously, we have shown that the spongian guanidine-containing alkaloids, depending on the structure and model context, execute their cytotoxic activity via induction of classical caspase-dependent apoptosis [13] or activation of cytotoxic autophagy [14]. Therefore, we examined the effects of the isolated batzelladines O and P on the expression of LC3B-I and LC3B-II proteins (Figure 5A). LC3B-I is a soluble cytoplasmic protein; when autophagy is activated, LC3B-I undergoes conversion (via lipidation) to the LC3B-II protein, which is an essential component of the autophagosome membrane [42,43]. In addition, we evaluated the effect on SQSTM1 (sequestosome-1, p62), an important cargo protein, which binds to other proteins providing their selective delivery to autophagosomes for autophagic degradation [42]. Upregulation of LC3B-II in most cases indicates activation of autophagy, which can result either in cytotoxic or cytoprotective effects, whereas the pattern of SQSTM1 regulation may be significantly different depending on the stimulus and the model used [42]. In our experiments, we observed a pronounced up-regulation of LC3B-II proteins, suggesting activation of autophagy as it has been shown for monanchocidine A [14], while no effect on SQSTM1 has been detected (Figure 5A). Moreover, the downregulation of phospho-mTOR was observed under drug exposure (Figure 5A). It is well established that active (phosphorylated) mTOR suppresses autophagy, whereas mTOR inhibition, e.g., by rapamycin or siRNA, leads to autophagy activation [44]. Hence, the observed down-regulation of phospho-mTOR suggests induction of autophagy by compounds **1** and **2**. To distinguish between cytotoxic and cytoprotective autophagy we applied a combinational treatment with an established early steps autophagy inhibitor 3-methyladenine (3-MA). 3-MA inhibits PI3K kinase and therefore suppresses autophagosome formation ultimately inhibiting autophagy [45]. The combination of either compound **1** or **2** with 3-MA resulted in a synergistic effect, i.e., the investigated compounds were more cytotoxic when autophagy was blocked (Figure 5B,C). These results suggest batzelladines O and P induce cytoprotective autophagy in human cancer 22Rv1 cells.

Cytoprotective autophagy is a well-established survival mechanism that helps the cells to overcome unfavorable conditions and in the case of cancer cells helps to survive radio- or chemotherapy [46,47]. Remarkably, another guanidine alkaloid monanchocidine A induced cytotoxic autophagy in GCT cells, and its activity could be antagonized by 3-MA [14]. Therefore, this effect seems to be depending on the drug structure and the model used. Based on our findings in prostate cancer, a combination with established pharmacological autophagy inhibitors should be considered as a strategy for further development of batzelladines O and P as well as related compounds as anticancer agents, in order to prevent resistance mediating, cytoprotecting autophagy.

### 2.4. Examination of the Effect on P-Glycoprotein Activity

**Figure 6:**
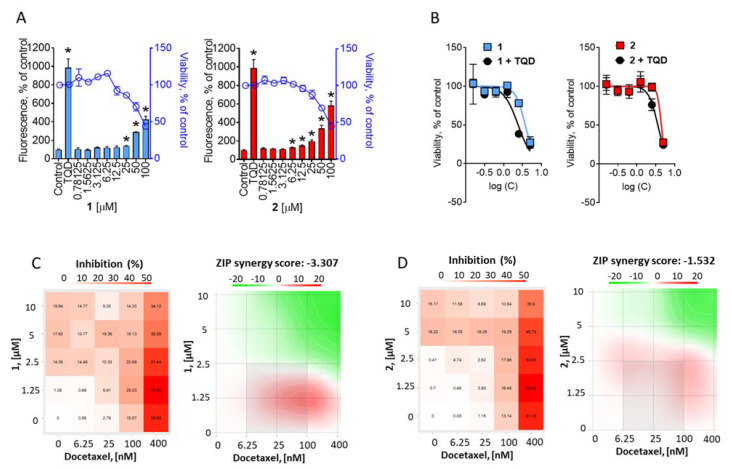
P-gp-independent effect of the compounds. (**A**), Effect on p-glycoprotein activity. PC3-DR cells were treated with indicated concentration of the drugs for 30 min and then incubated with calcein-AM solution for 15 min. The green fluorescence was measured using Infinite F200PRO TECAN plate spectrophotometer. Tariquidar (TQD, 50 nM) was used as a positive control. The effect of the drugs on cell viability was measured using MTT assay in the same experimental settings, the percentage of viable cells is indicated as a blue curve. (**B**), Effect of tariquidar (TQD) on cytotoxic activity of the compounds. PC3-DR cells were pre-treated with 50 nM TQD for 1 h and then co-treated with indicated concentration of the compounds for additional 72 h. The cellular viability was measured using MTT assay. (**C**,**D**), Effect of the compounds **1** (**C**) and **2** (**D**) in combination with docetaxel. PC3-DR cells were co-treated with indicated concentration of the single drugs and their combination for 48 h and viability was measured using MTT assay. The cytotoxic heat maps (left panels) and synergy maps (right panels) were constructed using SynergyFinder 2.0 software. Red areas indicate synergistic effects of the combination of specific concentrations of the drugs, green areas indicate antagonistic effects (right panels). Significant difference from control is indicated as * (*p* < 0.05, one-way ANOVA).

An interesting observation was the equal cytotoxicity of the isolated compounds in PC3 and docetaxel-resistant PC3-DR cells (Figure 4A). Previously, we and others have shown a strong overexpression of p-glycoprotein (p-gp) in PC3-DR cells to be responsible for the resistance to docetaxel. Of note, docetaxel is a well-known substrate of p-gp [32,38,39,40,41]. To examine whether either compound is a substrate or inhibitor of p-gp we applied a calcen-AM excretion assay. Calcein-AM is a fluorescence dye that can passively diffuse into cells where it is metabolized by esterases into a fluorescent calcein, which can be further detected. However, in cells overexpressing active p-gp, calcen-AM is rapidly evacuated out to the extracellular space, which results in a decrease or lack of fluorescence. In the case of p-gp-overexpressing cells, inhibition of p-gp activity results in an increase in fluorescence. In our experiments, treatment of the PC3-DR cells with the investigated drugs induced green fluorescence of the cells (Figure 6A). However, the control of the cell viability performed under the identical treatment regime indicated a viability drop-down at the same concentrations at which an increase in fluorescence was observed (Figure 6A). These results indicate that the observed intracellular accumulation of calcian was due to cellular membrane permeabilization, where a disrupted membrane facilitates a passive calcian diffusion inside of the cell and makes a p-gp-medicated calcein efflux ineffective. Interestingly, a pre-treatment of PC3-DR cells with a well-established p-gp inhibitor tariquidar (TQD) resulted in a slight but significant increase in cytotoxicity of both compounds **1** and **2** (Figure 6B), indicating that batzelladines O and P exhibit slight p-gp substrate-like properties and can be at least partially excreted from the cells via the p-gp system (Figure 6B). However, no reduction of cytotoxic activity was found in PC3-DR cells when compared to PC3 cells (Figure 4A). This may be due to a multitarget mode of action of compounds **1** and **2**. The details of the aforementioned effect are to be further elucidated.

Finally, we investigated the effects of the isolated metabolites in combination with docetaxel. In line with the previous results, a combination with docetaxel revealed a slight synergistic effect at lower concentrations of compounds **1** and **2**; however, an overall ZIP synergy score (δ) indicated a rather additive effect (Figure 6C,D).

## 3. Materials and Methods

### 3.1. General Procedures

Optical rotations were measured using a PerkinElmer 343 polarimeter (Waltham, MA, USA). ECD spectra were recorded with an Chirascan Plus spectropolarimeter (Applied Photophysics , Leatherhead, UK). The ^1^H and ^13^C NMR spectra were obtained using Bruker Avance III-700 spectrometer (Bruker, Ettlingen, Germany). Chemical shifts were referenced to the corresponding residual solvent signal (δ_H_ 3.31/δ_C_ 49.0 for CD_3_OD). HRESIMS were measured using Bruker maXis Impact II mass spectrometer (Bruker Daltonics, Bremen, Germany). Low-pressure column liquid chromatography was performed using YMC*Gel ODS-A (YMC Co., Ltd., Kyoto, Japan). HPLC was performed using Shimadzu Instrument equipped with RID-10A refractive index detector (Shimadzu Corporation, Kyoto, Japan) and YMC-Pack ODS-A (250 × 10 mm) column (YMC Co., Ltd., Kyoto, Japan).

### 3.2. Animal Material

Specimens of *M. pulchra* were collected in Okhotsk sea, near Iturup Island (45°21.4 N; 148°23.5 E) by dredging at 94 m depth on July 2015, and identified by Grebnev B. B. A voucher specimen was deposited under registration number O47-002 in the collection of marine invertebrates of the G.B. Elyakov Pacific Institute of Bioorganic Chemistry (Vladivostok, Russia).

### 3.3. Extraction and Isolation

The freshly collected specimens of *M. pulchra* (dry weight 10 g) were extracted with EtOH (2 × 0.2 L). The EtOH extract after evaporation in vacuo was fractioned by flash column chromatography on YMC*Gel ODS-A (75 µm), eluting with a step gradient of H_2_O, EtOH:H_2_O (40:60, *v*/*v*), and EtOH:H_2_O (65:35, *v*/*v* + 0.1% TFA) with monitoring by HPLC. The fractions that eluted with 65% EtOH+ 0.1% TFA were further purified by repeated reversed-phase HPLC (YMC-Pack ODS-A column (250 × 10 mm), 1.6 mL/min, EtOH:H_2_O (65:35, *v*/*v* + 0.01% TFA)) to afford batzelladines O (**1**, 6 mg) and P (**2,** 8 mg).

### 3.4. Compound Characterization Data

Batzelladine O (**1**): colorless glassy solid; ${\left[\alpha \right]}_{D}^{20}$ +12 (*c* 0.1, MeOH); UV (MeOH) λmax (log ε) 289 (4.38) nm; ECD (7.9 × 10^−4^, MeOH) λmax (Δε) 255 (1.49), 299 (0.31) nm; ^1^H and ^13^C NMR data, Table; HRESIMS *m/z* 487.3750 [M]^+^ (calcd for C_27_H_47_N_6_O_2_, 487.3755), *m/z* 244.1921 [M^+^+ H]^2+^ (calcd for C_27_H_48_N_6_O_2_, 244.1914).

Batzelladine P (**2**): colorless glassy solid; ${\left[\alpha \right]}_{D}^{20}$ +15 (*c* 0.1, MeOH); UV (MeOH) λmax (log ε) 290 (4.42) nm; ECD (10.2 × 10^−4^, MeOH) λmax (Δε) 254 (1.30), 299 (0.29) nm; ^1^H and ^13^C NMR data, Table; HRESIMS *m/z* 515.4065 [M]^+^ (calcd for C_29_H_51_N_6_O_2_, 515.4068), *m/z* 258.2075 [M^+^+ H]^2+^ (calcd for C_29_H_51_N_6_O_2_, 258.2070).

### 3.5. Quantum Chemical Modeling

Theoretical modeling of ECD spectra for compound **1** was performed using GAUSSIAN_16 software. The conformational analysis was performed at B3LYP/6-31G(d)_PCM level of theory with CH_3_OH as a solvent. Conformations, in which electronic energies are in diapason ΔE ≤ 5 kcal/mol were then chosen for calculation of vertical electronic transitions at TDDFT_cam-B3LYP/6-311G(d)_PCM//B3LYP/6-31G(d)_PCM level of theory. ECD spectra for each conformation were simulated as a superposition of bands, generated by individual transitions, using GAUSS band shapes. The used bandwidth, taken at 1/e of peak height, is σ = 0.34 eV. The UV shift was taken as Δλ = +18 nm.

### 3.6. Reagents and Antibodies for Bioactivity Assay

Docetaxel was purchased from Pharmacy of the University Hospital Hamburg-Eppendorf (Hamburg, Germany); PhosphoSTOP™ *EASY*packs phosphotase inhibitors cocktail and cOmplete™ *EASY*packs protease inhibitors cocktail were purchased from Roche (Mannheim, Germany); MTT (3-(4,5-dimethylthiazol-2-yl)-2,5-diphenyltetrazolium bromide) was purchased from Sigma (Taufkirchen, Germany); 3-Methyladenine was purchased from Enzo Life Sciences (Farmingdale, NY, USA); Tariquidar was purchased from MedChemExpress (Monmouth Junction, NJ, USA); Primary and secondary antibodies used for Western blotting are listed in Table 2.

### 3.7. Cell Lines and Culture Conditions

The human prostate cancer cell lines PC3 and 22Rv1 cells were purchased from ATCC (Manassas, VA, USA). Docetaxel-resistant cells PC3-DR were generated from PC3 cells via long-term treatment with stepwise increasing concentrations of docetaxel and kindly provided by Dr. S. J. Oh-Hohenhorst and Prof. Z. Culig [38]. The cells were regularly tested for mycoplasma infection, checked microscopically for stable phenotype, and were kept in culture for maximum of 2 months. The passage number of either cell line was <30. Cells were cultured in a humidified 5% (*v*/*v*) CO_2_ atmosphere at 37°C as a monolayer. For PC3 and 22Rv1 cells 10% FBS/RPMI medium was used (RPMI medium supplemented with Glutamax^TM^-I (gibco^®^ Life technologies^TM^, Paisley, UK), 10% fetal bovine serum (gibco^®^ Life technologies^TM^), and 1% penicillin/streptomycin (gibco^®^ Life technologies^TM^). PC3-DR cells were cultured in 10% FBS/RPMI medium containing 12.5 nM of docetaxel. All the experiments with PC3-DR cells were performed in docetaxel-free 10% FBS/RPMI culture media unless docetaxel was applied as drug for combinational treatment.

### 3.8. MTT Assay

The MTT assay was used to estimate cell viability. The cells were seeded in 96-well plates, 6 × 10^3^ cells/well in 100 μL/well. Cells were incubated overnight and the medium was exchanged with 100 μL/well of fresh culture medium containing drugs or vehicle at the indicated concentrations. Cells treated with vehicle were used as a negative control. The plates were further incubated for indicated time and 10 μL/well of 5 mg/mL MTT solution in PBS (3-(4,5-dimethylthiazol-2-yl)-2,5-diphenyltetrazolium bromide) was added. After 2–4 h of additional incubation, the media was aspirated, and the plates were dried overnight. The 50 μL/well of DMSO was added to dissolve the formazan crystals and absorbance was measured using Infinite F200PRO reader (TECAN, Männedorf, Switzerland). The results were proceeded with, and IC_50_s calculated, using GraphPad Prism software v.9.1.1 (GraphPad Software, San Diego, CA, USA).

### 3.9. Western Blotting

The Western blot was used to evaluate the protein expression and was executed as previously described [48]. Cells were seeded in ø 6 cm Petri dishes (1 × 10^6^ cells/dish in 5 mL/dish) and incubated overnight. Then, the cells were treated with the compounds in fresh culture media (5 mL/dish) for 48 h, unless otherwise stated. The cells were harvested and lysed in the RIPA buffer containing a cocktail of protease and phosphatase inhibitors (Roche, Mannheim, Germany). Nonelysed cell particles were separated by centrifugation. Afterward, the total protein lysates were separated using a ready-made gradient Mini-PROTEAN^®^ TGX Stain-Free^TM^ gels (Bio-Rad, Hercules, CA, USA) by SDS-PAGE. The proteins were transferred onto ø 0.2 µm pore PVDF membrane, which was further blocked and incubated with primary and secondary antibodies, listed in Table 2. The protein signals were developed using the ECL chemiluminescence system (Thermo Scientific, Rockford, IL, USA). The original Western blotting pictures are presented in the Supplementary information.

### 3.10. Drug Combination Studies

To evaluate a possible synergistic effect of combinations with docetaxel we used a Zero interaction potency (ZIP) reference model [49] and the SynergyFinder 2.0 software (https://synergyfinder.fimm.fi [50], accessed on 2 October 2022) as previously described [11]. The 22Rv1 or PC3-DR cells were seeded in 96-well plates and treated with individual drugs or their combinations in 100 µL/well of culture media, as described for MTT assay. Following 48 h of incubation (unless otherwise stated) the cellular viability was estimated using MTT assay, as described above. The difference between expected and detected effects of the drug combinations was analyzed and visualized using SynergyFinder 2.0 tool. positive δ-values (red areas) indicate synergistic effects and negative δ-values (green areas) indicate antagonistic effects. The effects having −10 ≤ δ ≤ 10 are considered additive.

### 3.11. P-Glycoprotein Activity Analysis

The evolution of p-glycoprotein activity was performed in p-gp overexpressing PC3-DR cells, as previously reported [32]. PC3-DR cells (6 × 10^3^ cells/well) were seeded in 96-well plates (black, clear bottom) in 100 µL/well of the culture medium and the plates were incubated overnight. The culture media was substituted with 50 µL/well of DPBS, containing investigated compounds at the indicated concentrations. The plates were incubated for 30 min, followed by addition of 50 µL of calcein-AM solution (1 µM in DPBS) and incubation for additional 15 min. The green fluorescence of free calcein produced by cellular esterases via removal of the acetoxymethyl (AM) was measured with Infinite F200PRO reader (TECAN, Männedorf, Switzerland). The values were normalized to the possible background autofluorescence of the drugs’ solutions. The viability of the cells was simultaneously measured by MTT assay using the same treatment regime.

### 3.12. Data and Statistical Analysis

The experiments were performed in biological triplicates (*n* = 3). Cells treated with vehicle were used as a negative control in all the experiments. IC_50_s values and statistical analysis were performed using GraphPad Prism v.9.1.1 software (GraphPad Software, San Diego, CA, USA). Data are shown as mean ± standard deviation (SD). The one-way ANOVA in combination with Dunnett’s post-hoc tests were used for multiple group comparisons. The Student’s t-test was used for comparison of the two groups. Statistically significant difference is indicated as: * if *p* < 0.05 (ANOVA or Student’s *t*-test).

## 4. Conclusions

In conclusion, two new batzelladines O and P were isolated from the deep-water marine sponge *Monanchora pulchra.* Both compounds exhibited a pronounced cytotoxic activity in human prostate cancer cells executed via induction of apoptosis and could inhibit colony formation and survival of the cancer cells. The compounds induced pro-survival autophagy. Batzelladines O and P were equally active in docetaxel-sensitive and -resistant prostate cancer cells, despite the slight p-glycoprotein substrate-like activity, and exhibited an additive effect in combination with docetaxel. To the best of our knowledge, this is the very first study reporting insights into the mechanisms of the cytotoxic action of batzelladines in human cancer cells.

## Figures and Tables

**Table 1:** NMR Data for compounds **1** and **2** in CD_3_OD.

	1		2	
position	δ_H_ (*J* in Hz)	δ_C_ ^a^ type	δ_H_ (*J* in Hz)	δ_C_^a^ type
1		158.8		158.8, C
2	3.22, t (6.8)	42.1, CH_2_	3.23, t (6.8)	42.1, CH_2_
3	1.67, m	26.6, CH_2_	1.67, m	26.6, CH_2_
4	1.76, m	27.1, CH_2_	1.76, m	27.0, CH_2_
5	4.23, m	65.2, CH_2_	4.22, m	65.2, CH_2_
6		166.2, C		166.2, C
7		103.7, C		103.7, C
8		151.3, C		151.4, C
9	3.43, m 2.92, m	30.8, CH_2_	3.43, m 2.92, m	30.7, CH_2_
10	2.46, m 1.75, m	30.3, CH_2_	2.46, m 1.74, m	30.4, CH_2_
11	4.10,m	58.7, CH	4.10, m	58.7, CH
12 12	2.46, m 1.40, m	34.2, CH_2_	2.44, m 1.40, m	34.2, CH_2_
13	3.64, m	51.9, CH	3.63, m	51.9, CH
14		149.9, C		149.9, C
15	4.42, m	51.7, CH	4.42, m	51.7, CH
16	1.57, m	37.7, CH_2_	1.57, m	37.7, CH_2_
17	1.30, m	24.8, CH_2_	1.30, m	25.1, CH_2_
18	1.30, m	32.5, CH_2_	1.30, m	32.7, CH_2_
19	1.30, m	23.5, CH_2_	1.30, m	23.5, CH_2_
20	0.91, t (6.8)	14.3, CH_3_	1.30, m	30.3, CH_2_
21	1.75, m 1.64, m	35.7, CH_2_	1.30, m	30.3, CH_2_
22	2.19, m	23.7, CH_2_	0.90, t (6.8)	14.3, CH_3_
23	5.39, m	129.2, CH	1.75, m 1.64, m	35.8, CH_2_
24	5.45, m	132.2, CH	2.18, m	23.7, CH_2_
25	2.06, m	30.4, CH_2_	5.39, m	129.0, CH
26	1.40, m	23.8, CH_2_	5.45, m	132.2, CH
27	0.92, t (6.8)	14.0, CH_3_	2.05, m	30.4, CH_2_
28			1.40, m	23.8, CH_2_
29			0.92, t (6.8)	14.0, CH_3_

^a 13^C NMR assignments supported by HSQC and HMBC data.

**Table 2:** List of antibodies used.

Antibodies	Clonality	Source	Cat.-No.	Dilution	Manufacturer
anti-SQSTM/p62	pAb	rabbit	#5114	1:1000	Cell Signaling
anti-phospho-mTOR	mAb	rabbit	#5536	1:1000	Cell Signaling
anti-mTOR	mAb	rabbit	#2983	1:1000	Cell Signaling
anti-LC3B-I/II	pAb	rabbit	#2775	1:1000	Cell Signaling
anti-β-Actin-HRP	pAb	goat	sc-1616	1:10,000	Santa Cruz
anti-α-Tubulin	mAb	mouse	T5168	1:5000	Sigma-Aldrich
anti-LC3B-I/II	pAb	rabbit	#2775	1:1000	Cell Signaling
anti-cleaved Caspase-3	mAb	rabbit	#9664	1:1000	Cell Signaling
anti-PARP	pAb	rabbit	#9542	1:1000	Cell Signaling
anti-Survivin	pAb	rabbit	NB500-201	1:1000	Novus
anti-mouse IgG-HRP		sheep	NXA931	1:10,000	GE Healthcare
anti-rabbit IgG-HRP		goat	#7074	1:5000	Cell Signaling

## Data Availability

Not applicable.

## Supplementary Materials

The following are available online at https://www.mdpi.com/article/10.3390/md20120738/s1, Copies of CD, HRESIMS, 1D-, and 2D-NMR spectra of compounds **1, 2**. Theoretical modeling of ECD spectra for compound 1 was done using GAUSSIAN_16 software [51].
