# Supplementary material for: New Guanidine Alkaloids Batzelladines O and P from the Marine Sponge Monanchora pulchra Induce Apoptosis and Autophagy in Prostate Cancer Cells
Source: Mar Drugs. 2022 Nov 25;20(12):738. doi: 10.3390/md20120738 (PMC9783649; doi:10.3390/md20120738)
Supplement: Supplementary file 1 [file marinedrugs-20-00738-s001.zip › marinedrugs-2007385-supplementary.pdf]

Supporting Information

New Guanidine Alkaloids Batzelladines O and P from the Marine Sponge Monanchora pulchra Induce Apoptosis and Autophagy in Prostate Cancer Cells

Sergey A. Dyshlovoy [1,2,3,*,†], Larisa K. Shubina [4,†], Tatyana N. Makarieva [4,*], Alla G. Guzii [4], Jessica Hauschild [1,2], Nadja Strewinsky [1], Dmitrii V. Berdyshev [4], Ekaterina K. Kudryashova [4], Alexander S. Menshov [4], Roman S. Popov [4], Pavel S. Dmitrenok [4], Markus Graefen [2], Carsten Bokemeyer [1] and Gunhild von Amsberg [1,2]

[1] Department of Oncology, Hematology and Bone Marrow Transplantation with Section Pneumology, Hubertus Wald-Tumorzentrum, University Medical Center Hamburg-Eppendorf, 20251 Hamburg, Germany
[2] Martini-Klinik, Prostate Cancer Center, University Hospital Hamburg-Eppendorf, 20251 Hamburg, Germany;
[3] Institute of High Technologies and Advanced Materials, Far Eastern Federal University, Vladivostok 690922, Russia
[4] G.B. Elyakov Pacific Institute of Bioorganic Chemistry, Far Eastern Branch of the Russian Academy of Sciences, Pr. 100-let Vladivostoku 159, 690022 Vladivostok, Russia

* Correspondence: s.dyshlovoy@uke.de (S.A.D.); makarieva@piboc.dvo.ru (T.N.M.)
† These authors contributed equally to this work

Content

Supporting Information......1
Figure S1. ^{1}H NMR spectrum of batzelladine O (1) in CD_3OD (700 MHz). 2
Figure S2. ^{13}C NMR spectrum of batzelladine O (1) in CD_3OD (175 MHz). 3
Figure S3. ^{1}H-^{1}H COSY spectrum of batzelladine O (1) in CD_3OD......4
Figure S4. HSQC spectrum of batzelladine O (1) in CD_3OD. 5
Figure S5. HMBC spectrum of batzelladine O (1) in CD_3OD. 6
Figure S6. ROESY of batzelladine O (1) in CD_3OD. 7
Figure S7. HRESIMS and MS/MS spectra of batzelladine O (1) (positive ion mode). 8
Figure S8. ^{1}H NMR spectrum of batzelladine P (2) in CD_3OD (700 MHz). 9
Figure S9. ^{13}C NMR spectrum of batzelladine P (2) in CD_3OD (175 MHz). 10
Figure S10. ^{1}H-^{1}H COSY spectrum of batzelladine P (2) in CD_3OD. 11
Figure S12. HMBC spectrum of batzelladine P (2) in CD_3OD......13
Figure S13. ROESY of batzelladine P (2) in CD_3OD. 14
Figure S14. HRESIMS and MS/MS spectra of batzelladine P (2) (positive ion mode). 15
Figure S15. Posible stereoisomers of 1. 16
Quantum-chemical modeling. 17
Conformational analysis......18
Original Western blotting images......20

Figure S1. ^{1}H NMR spectrum of batzelladine O (**1**) in CD_3OD (700 MHz).

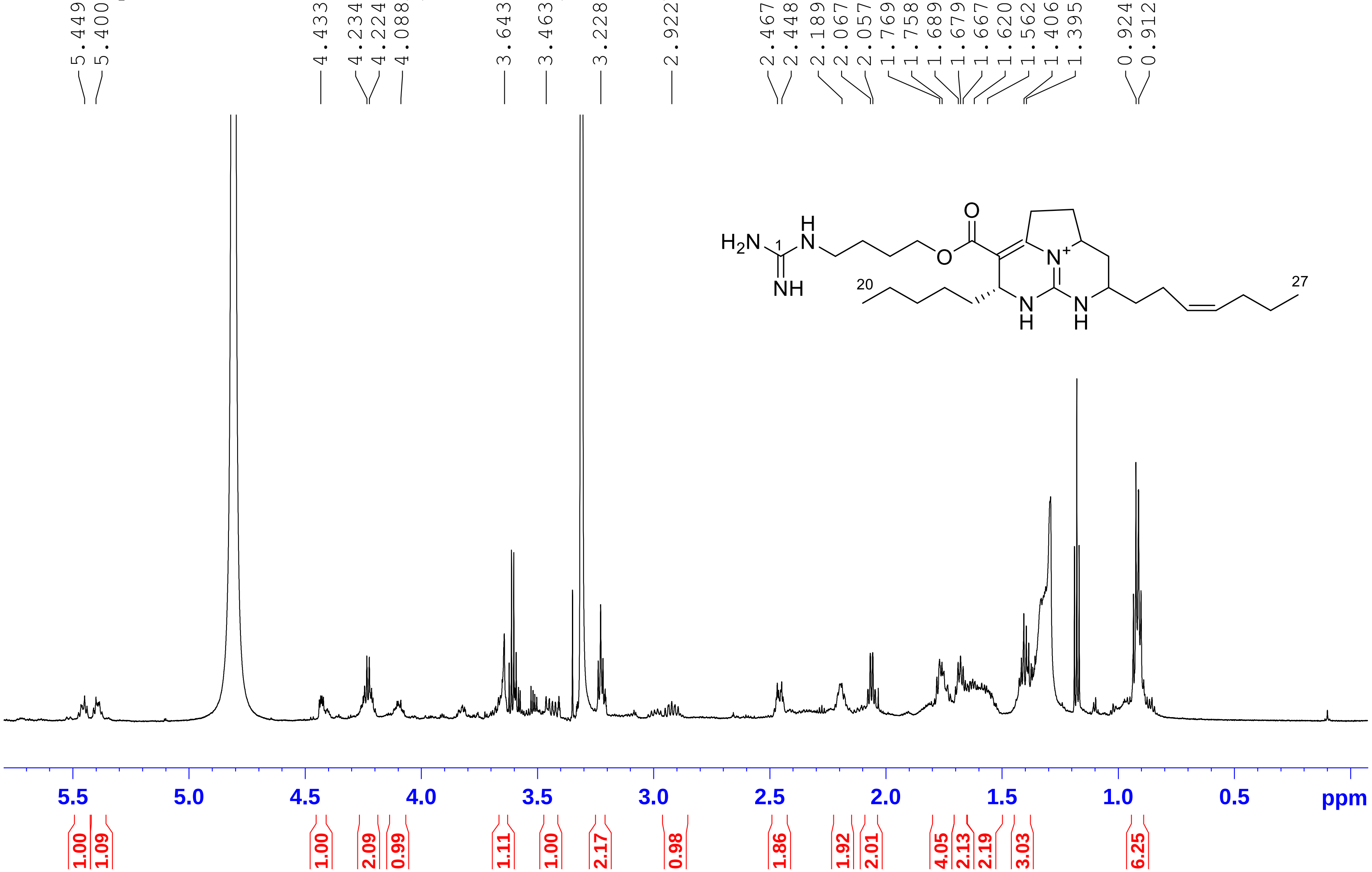

Figure S2. ^{13}C NMR spectrum of batzelladine O (**1**) in CD_3OD (175 MHz).

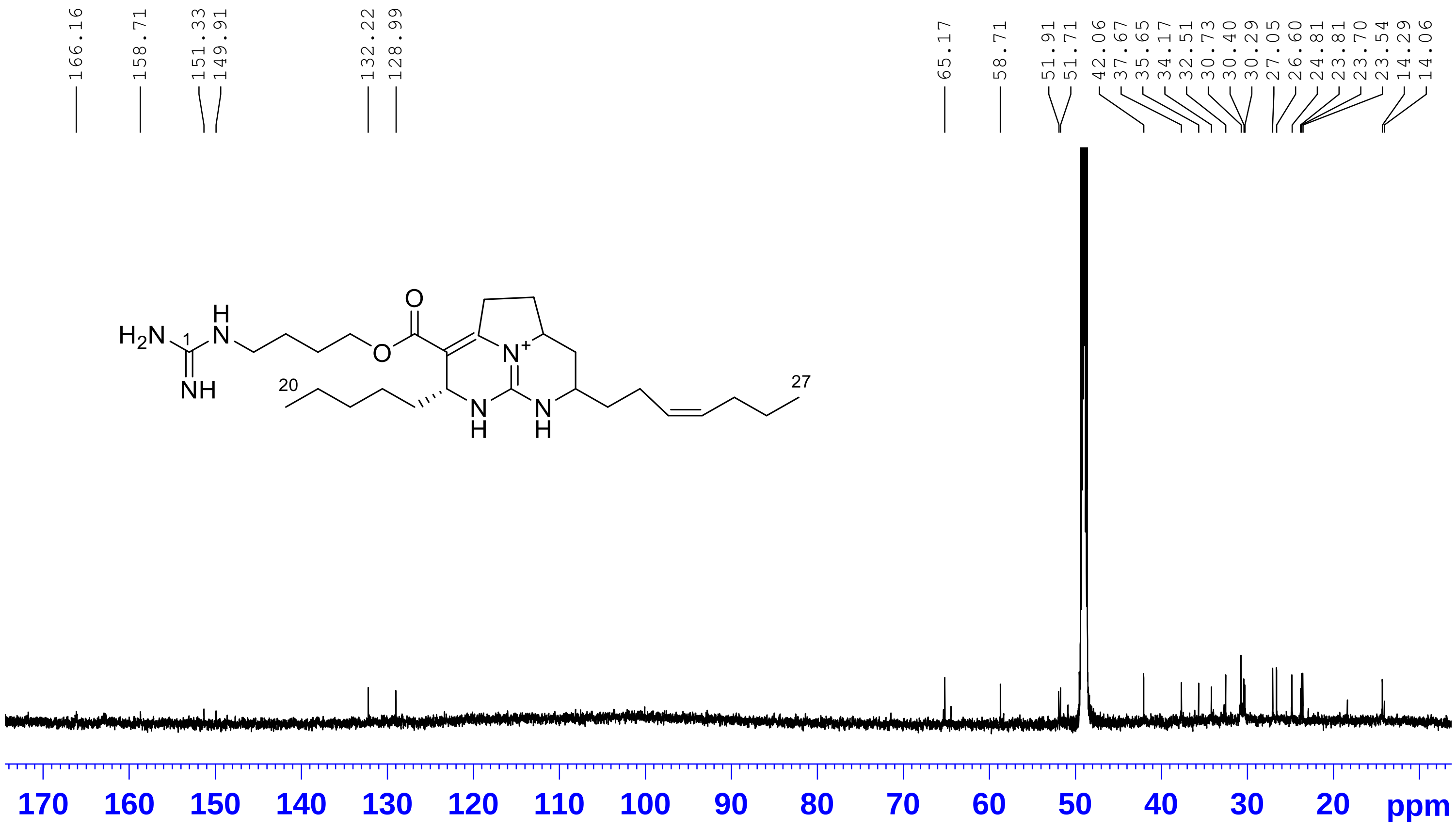

Figure S3. ^1H-^1H COSY spectrum of batzelladine O (**1**) in CD_3OD.

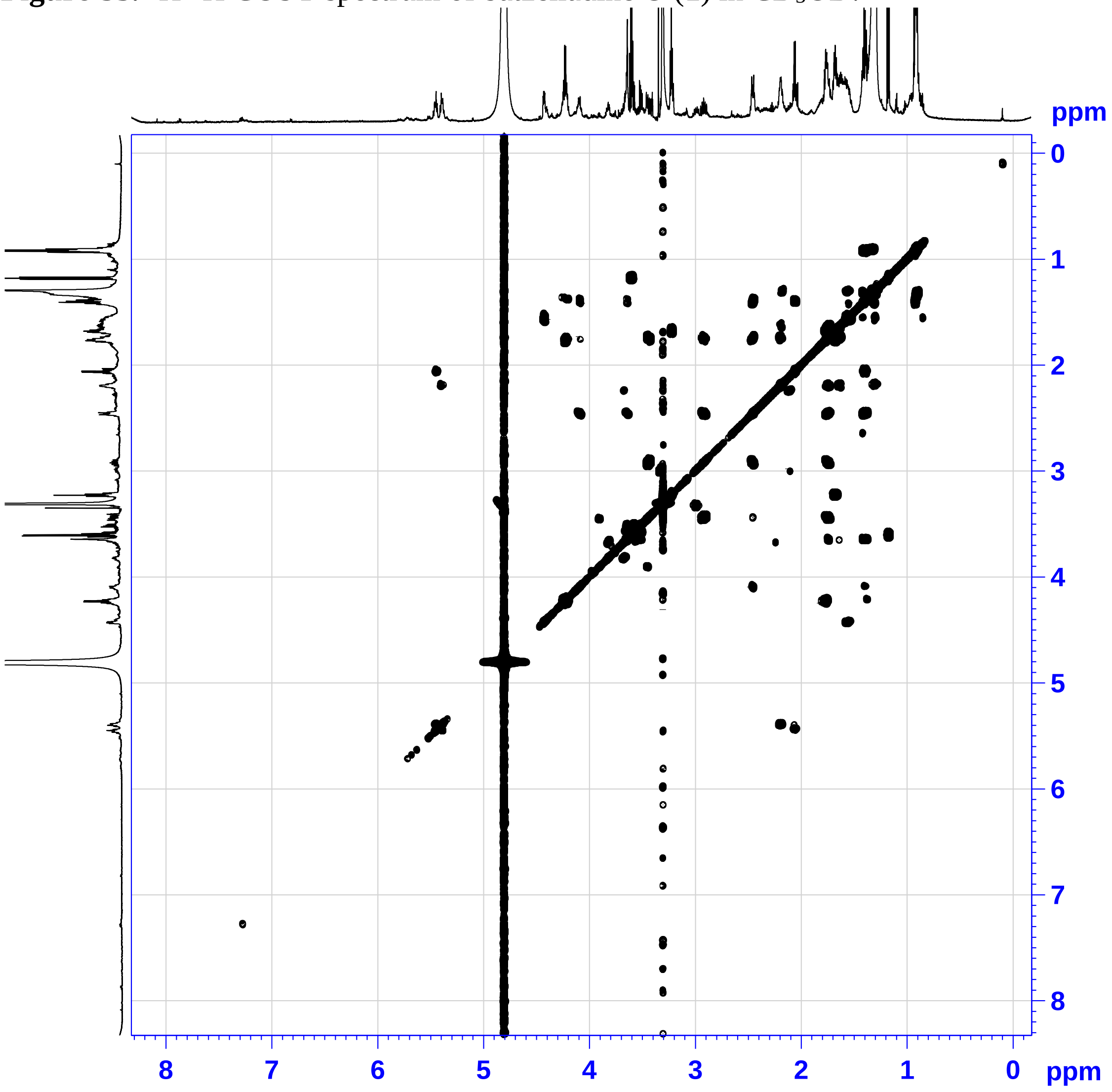

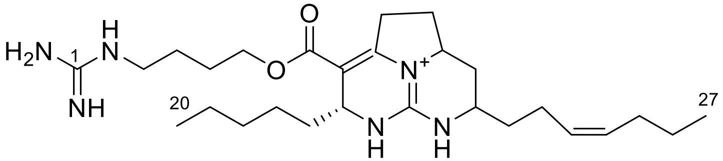

Figure S4. HSQC spectrum of batzelladine O (**1**) in CD_3OD.

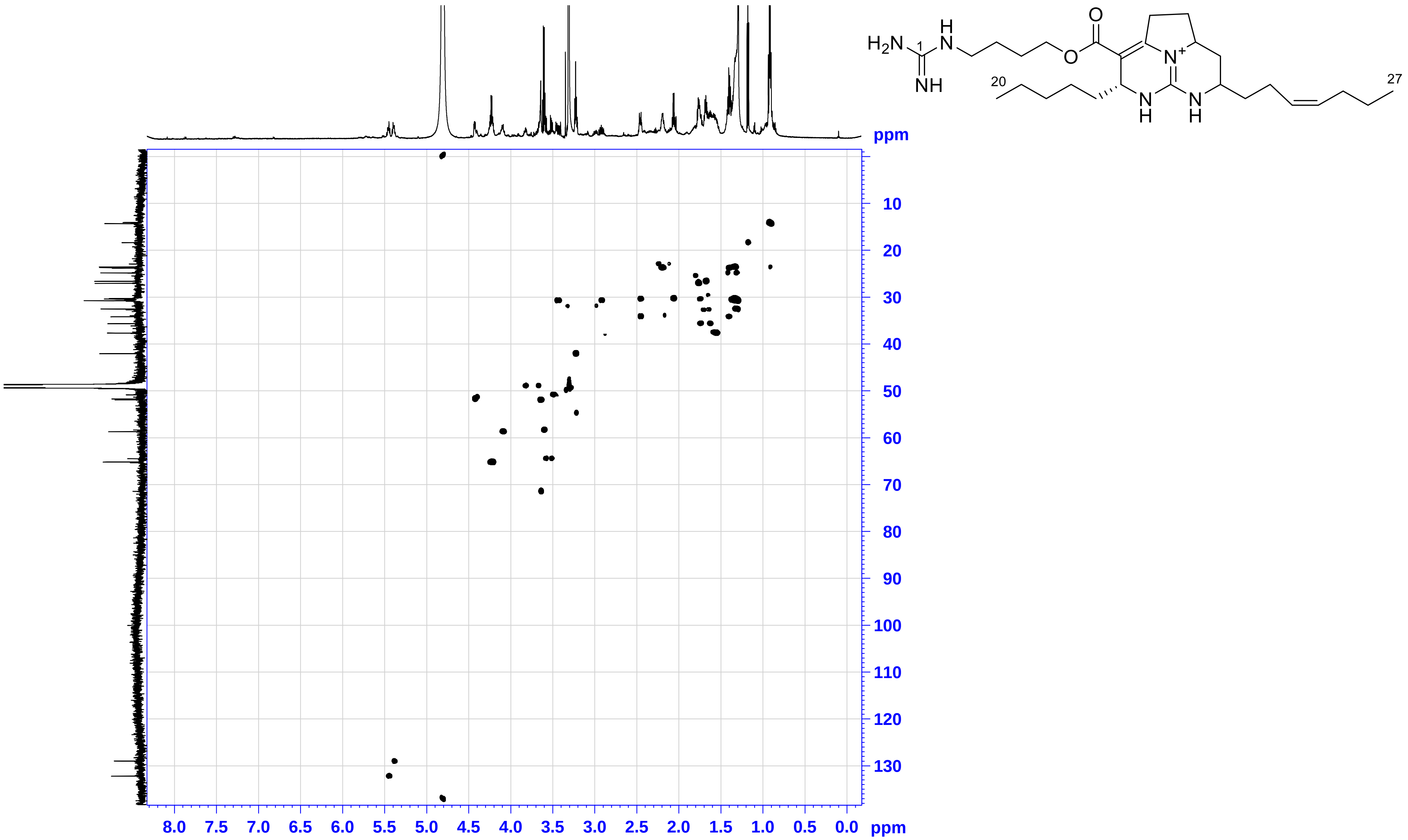

Figure S5. HMBC spectrum of batzelladine O (**1**) in CD_3OD.

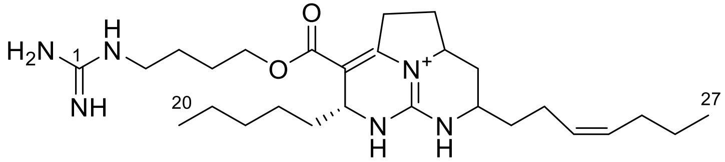

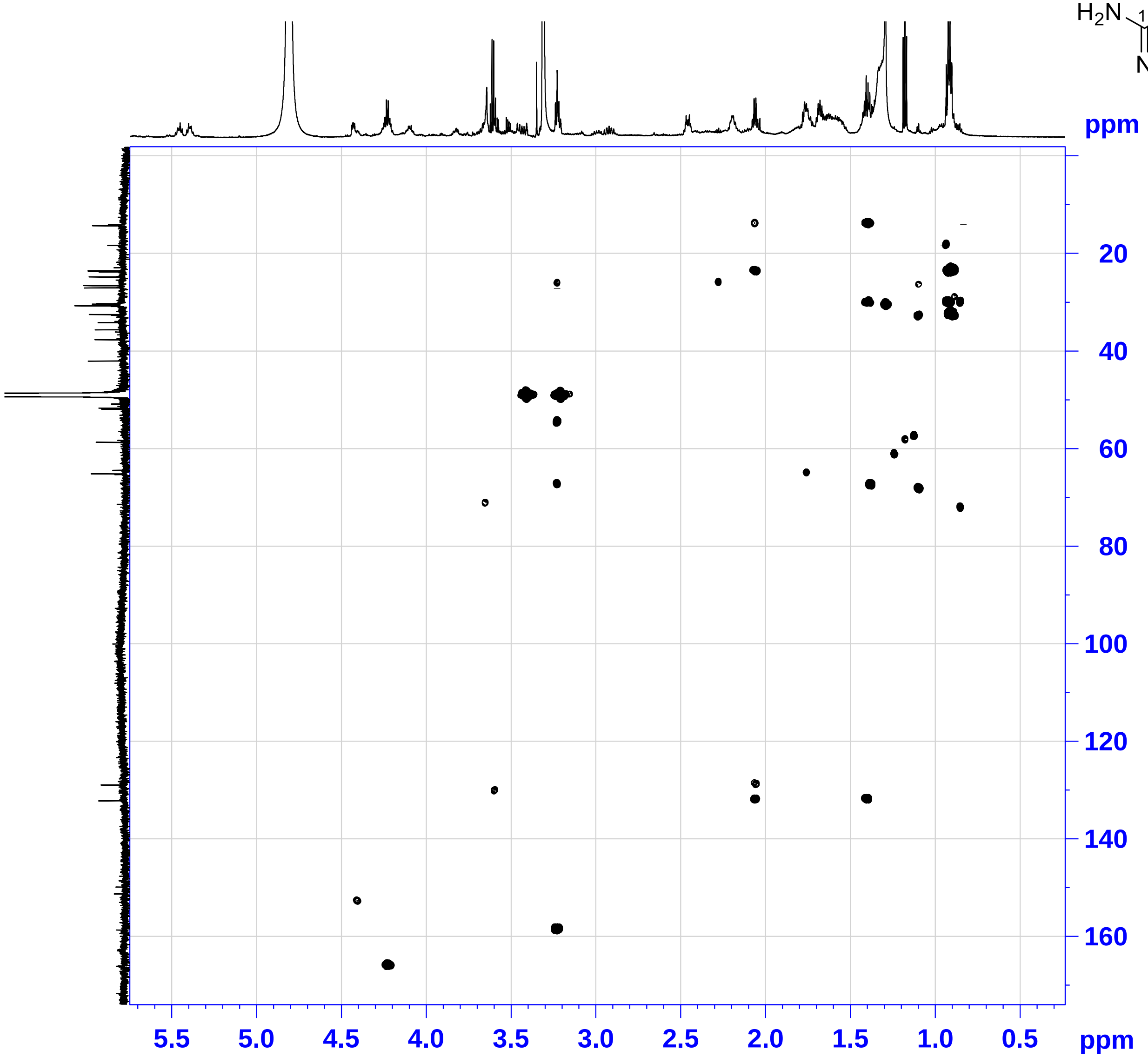

Figure S6. ROESY of batzelladine O (**1**) in CD3OD.

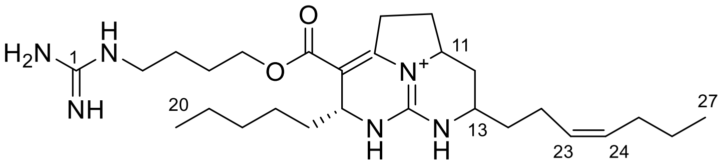

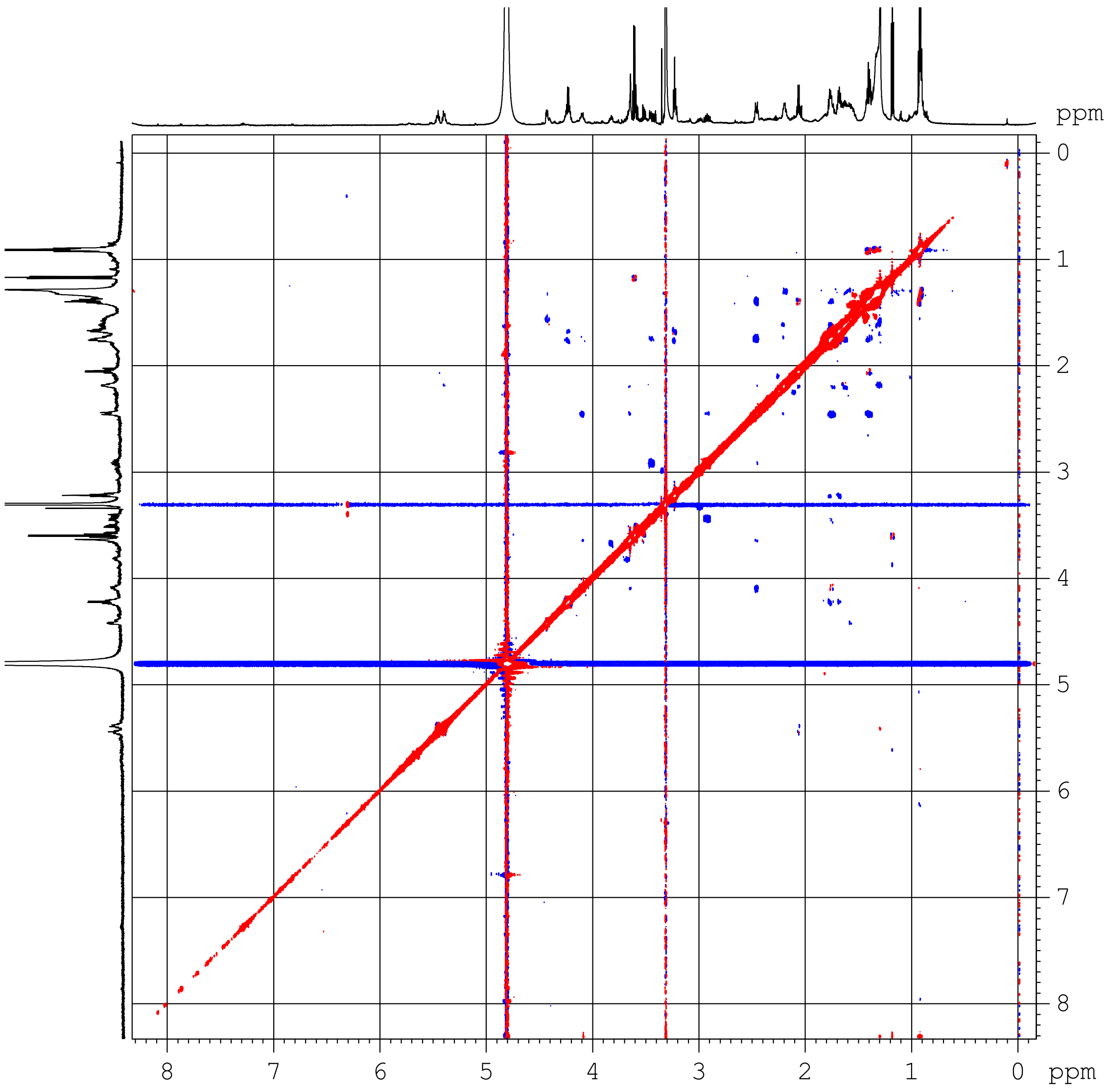

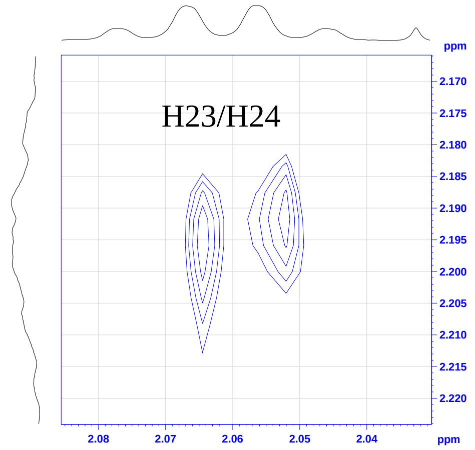

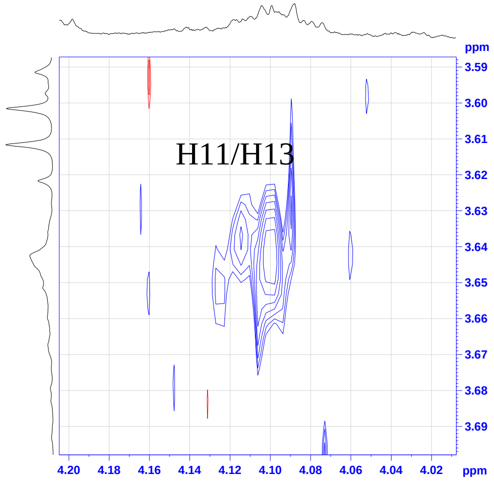

Figure S7. HRESIMS and MS/MS spectra of batzelladine O (**1**) (positive ion mode).

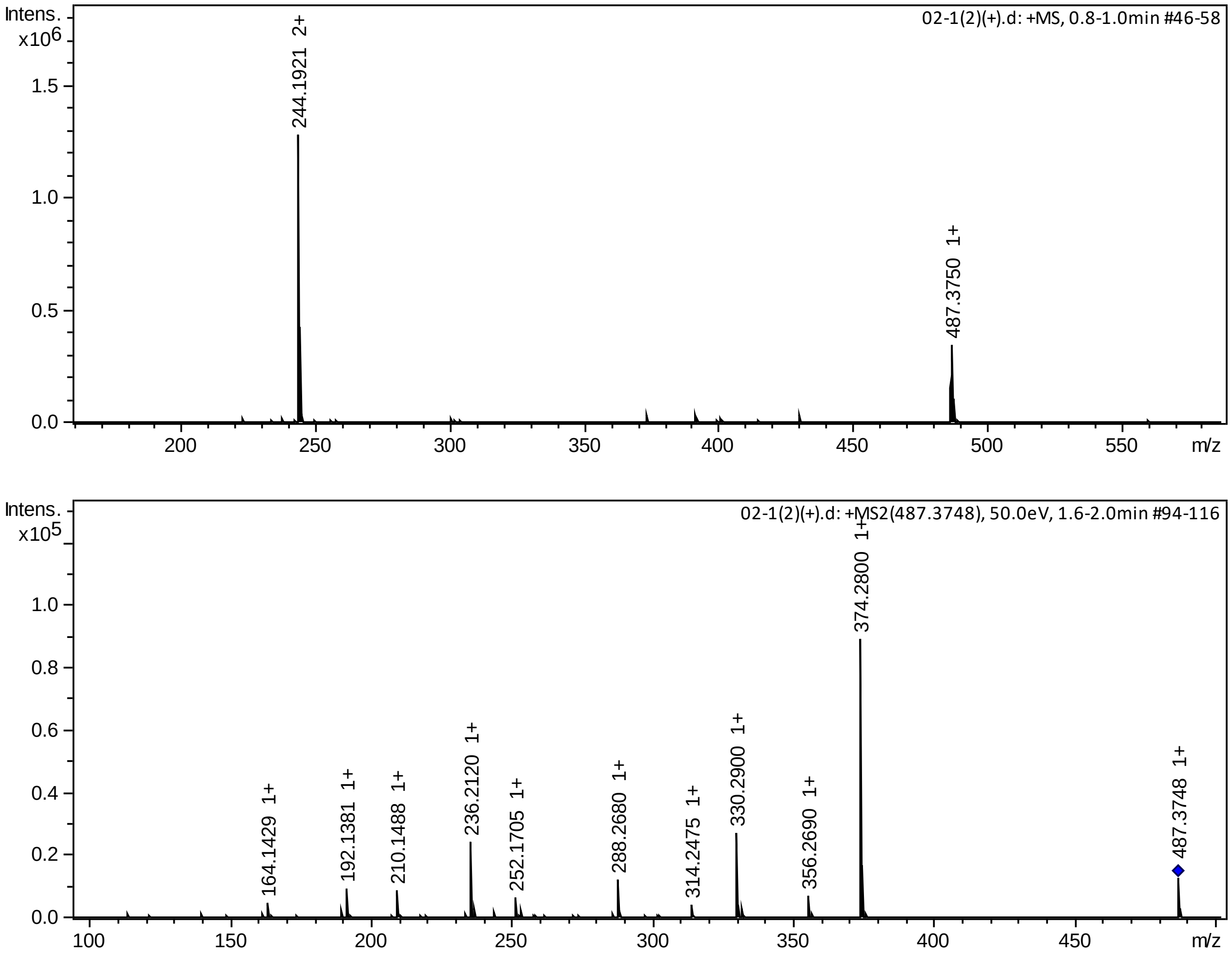

Figure S8. ^{1}H NMR spectrum of batzelladine P (**2**) in CD_3OD (700 MHz).

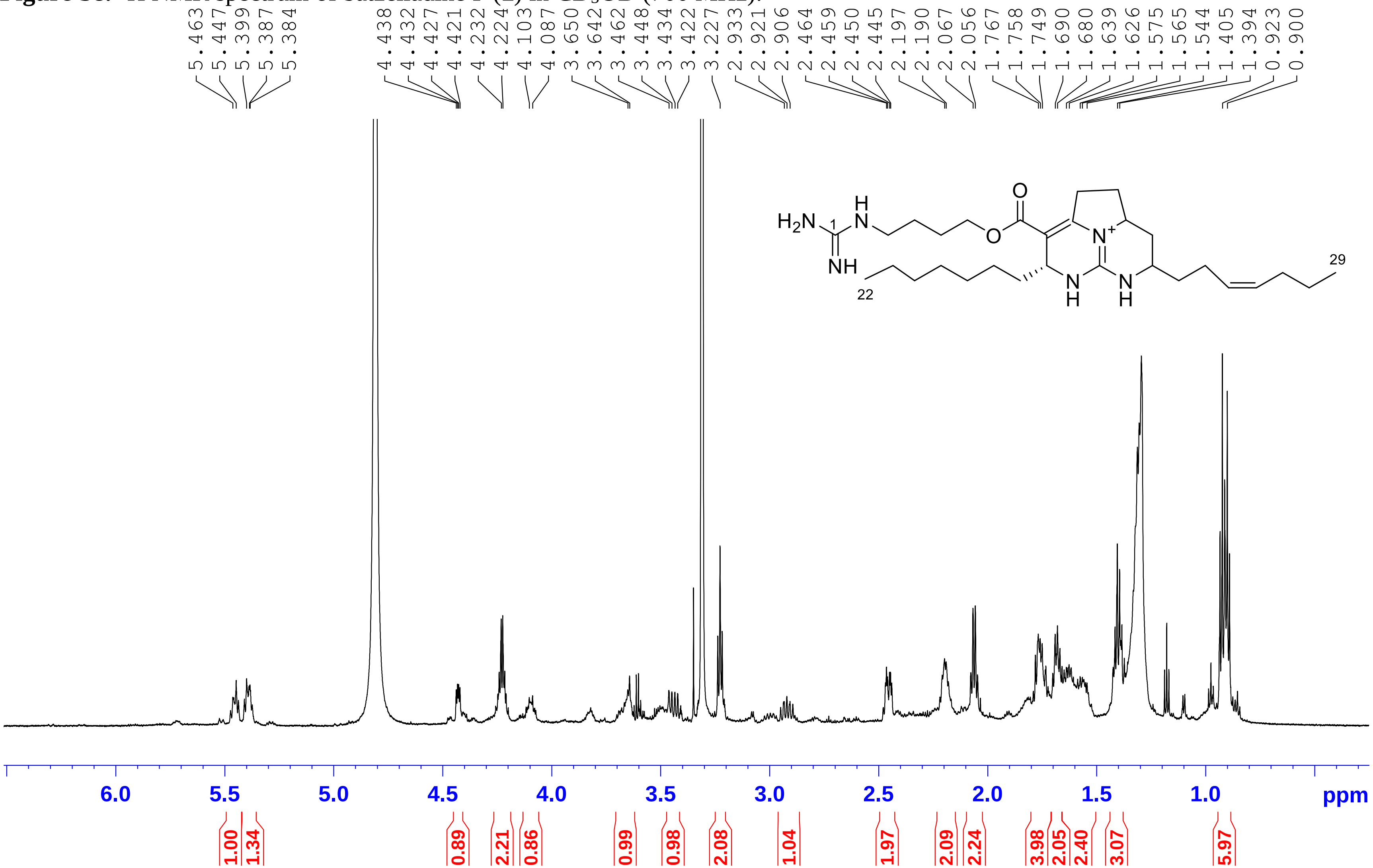

Figure S9. ^{13}C NMR spectrum of batzelladine P (**2**) in CD_3OD (175 MHz).

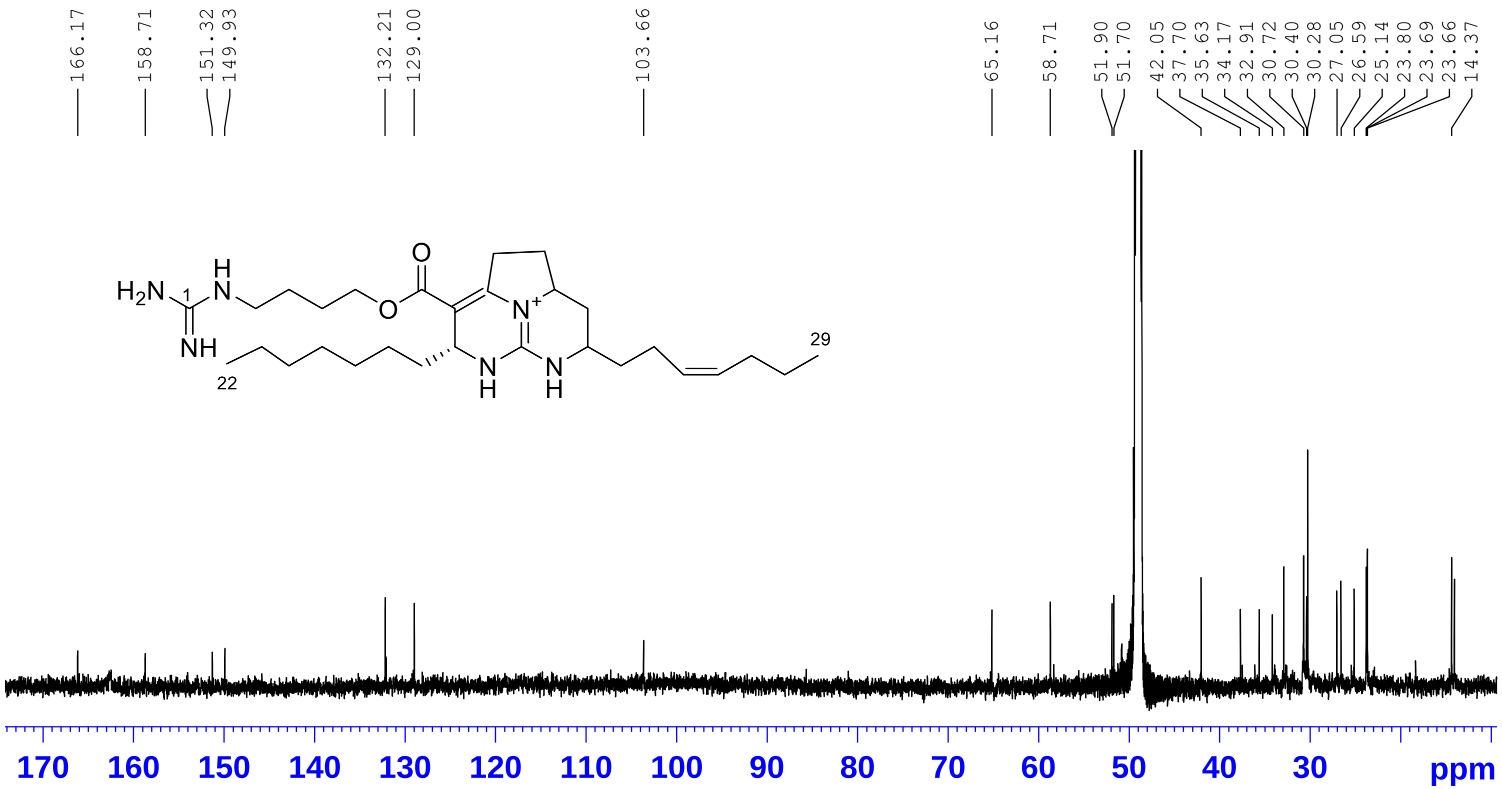

Figure S10. ¹H-¹H COSY spectrum of batzelladine P (**2**) in CD_3OD.

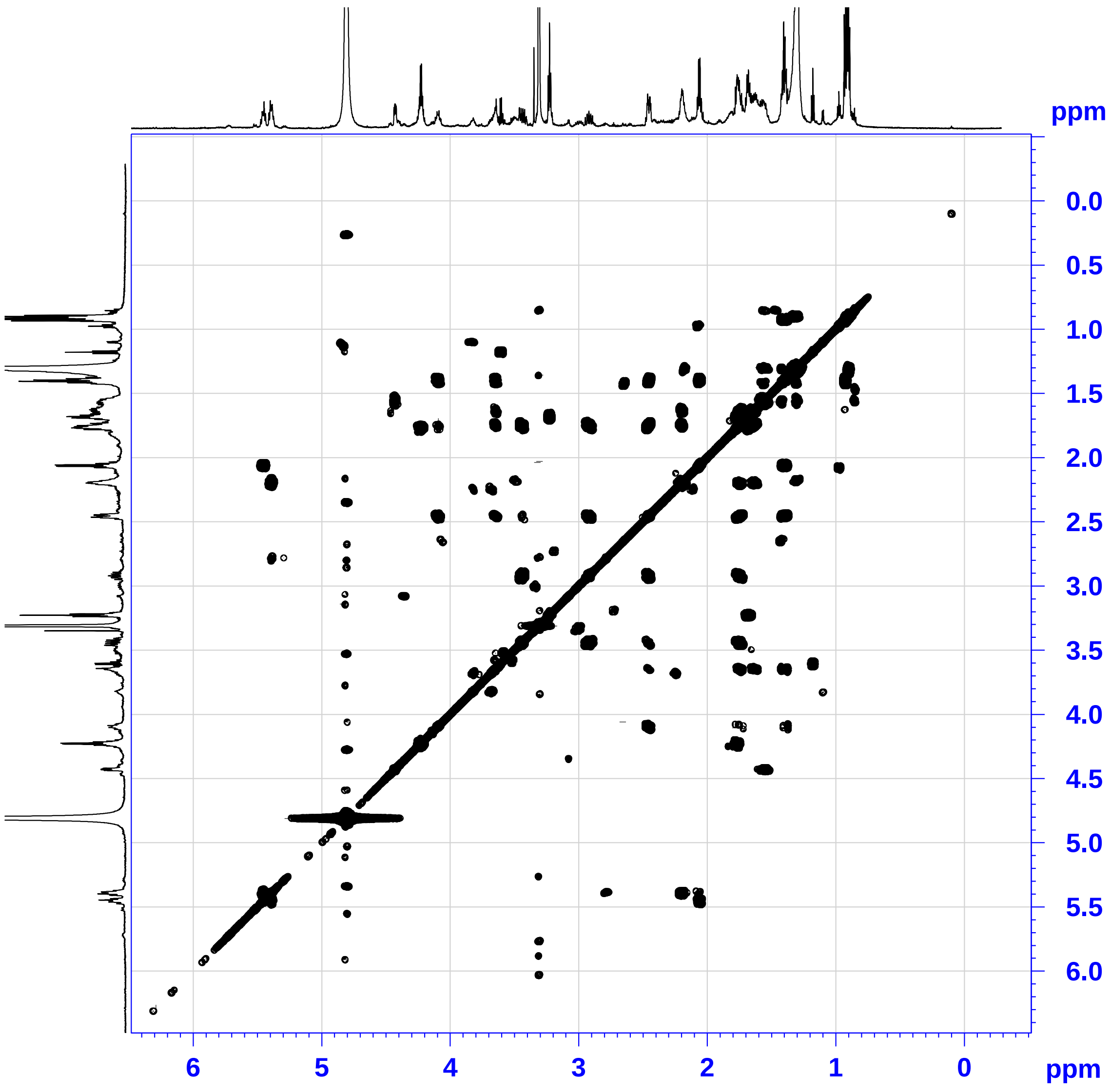

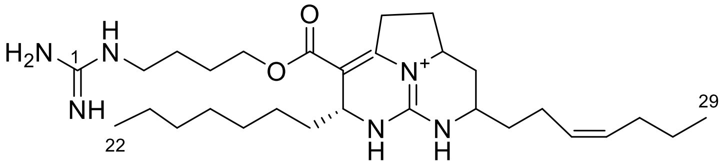

Figure S11. HSQC spectrum of batzelladine P (**2**) in CD3OD.

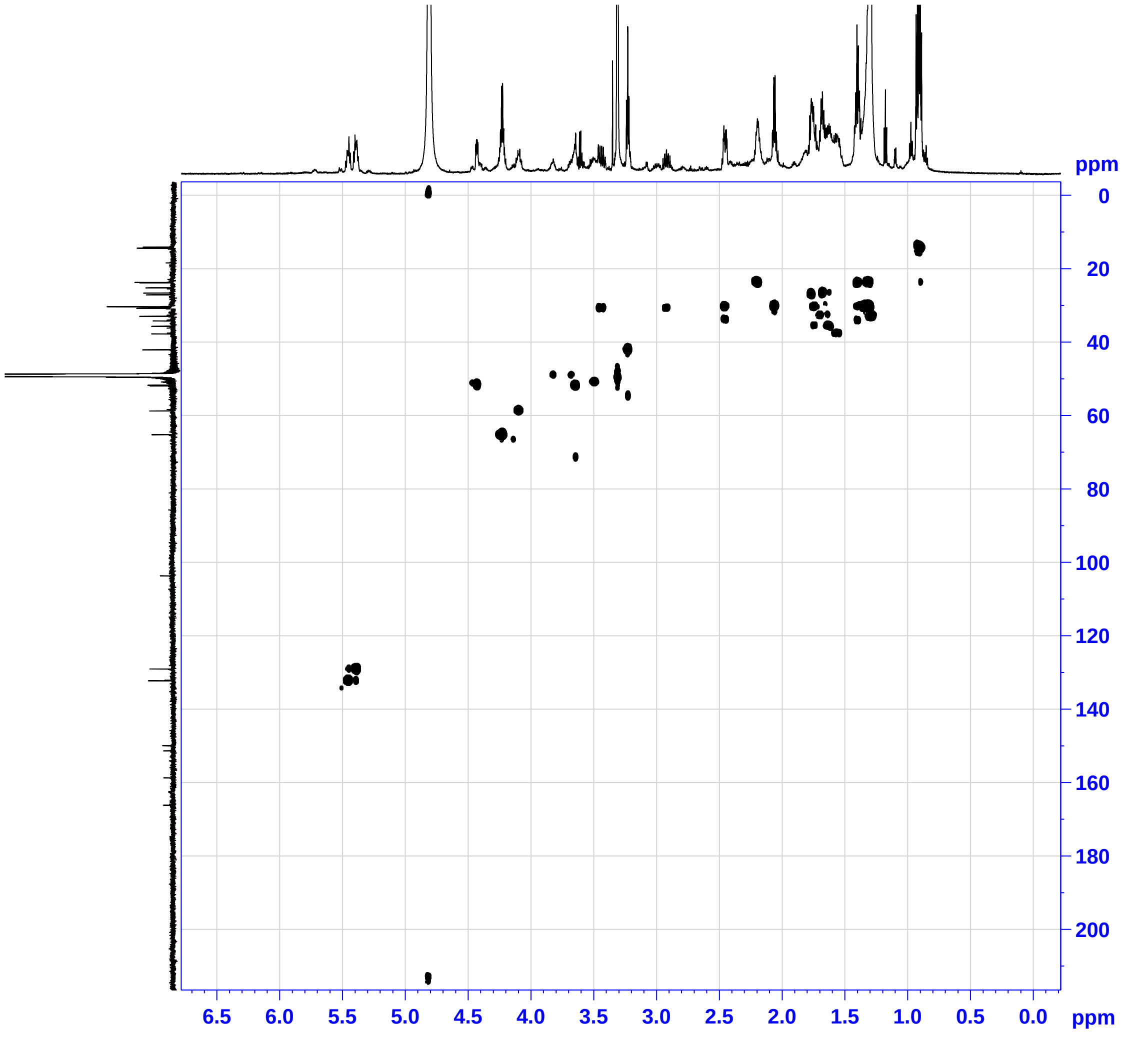

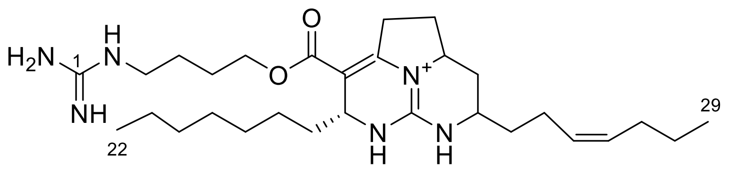

Figure S12. HMBC spectrum of batzelladine P (**2**) in CD_3OD.

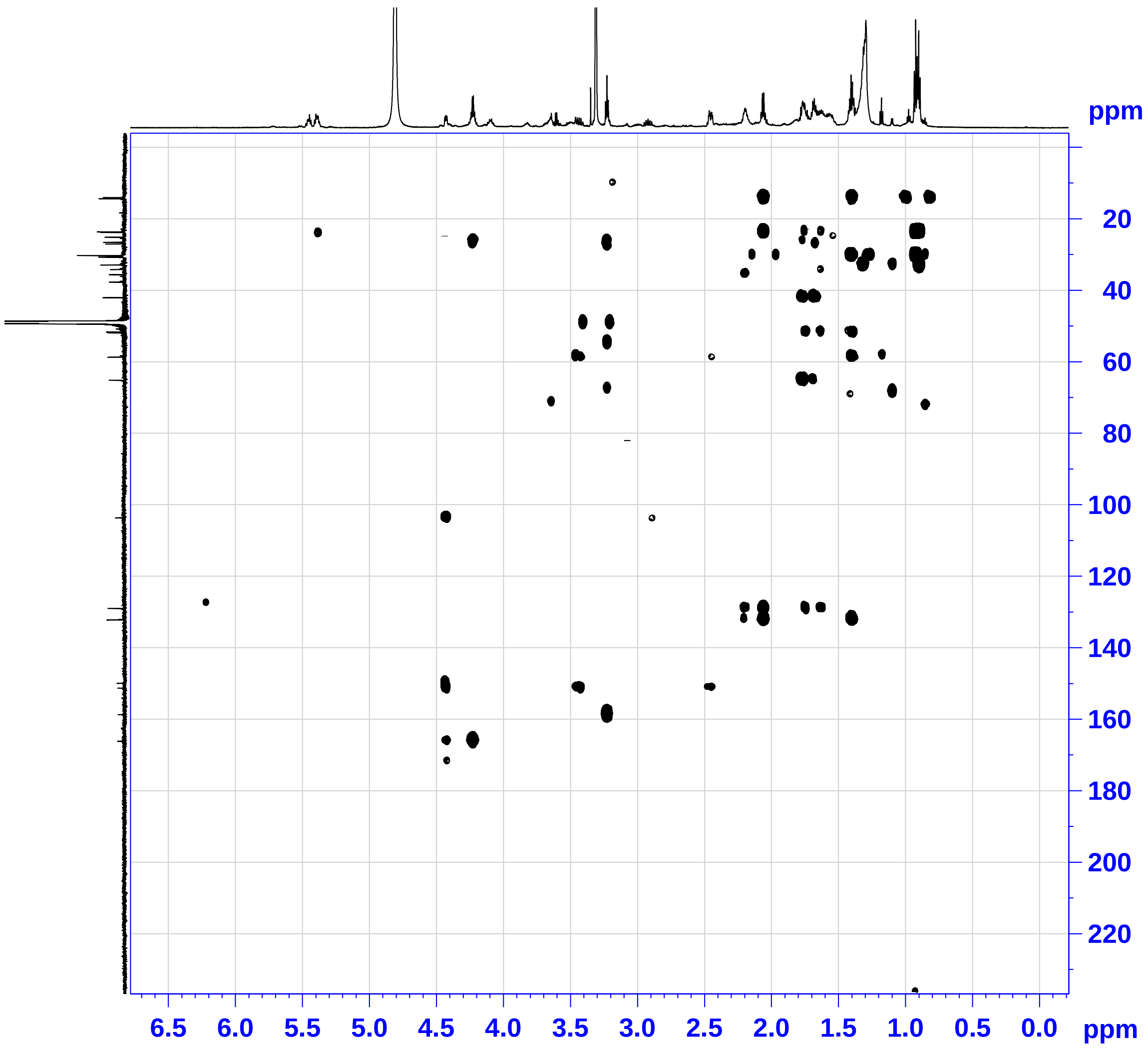

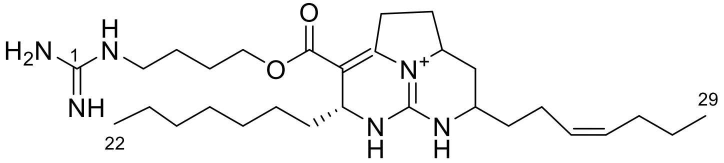

Figure S13. ROESY of batzelladine P (**2**) in CD3OD.

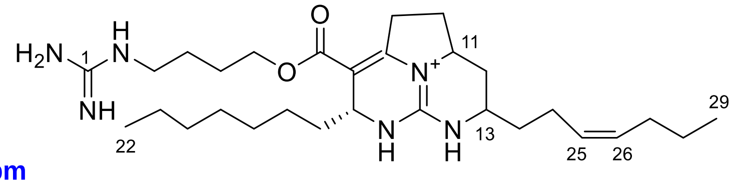

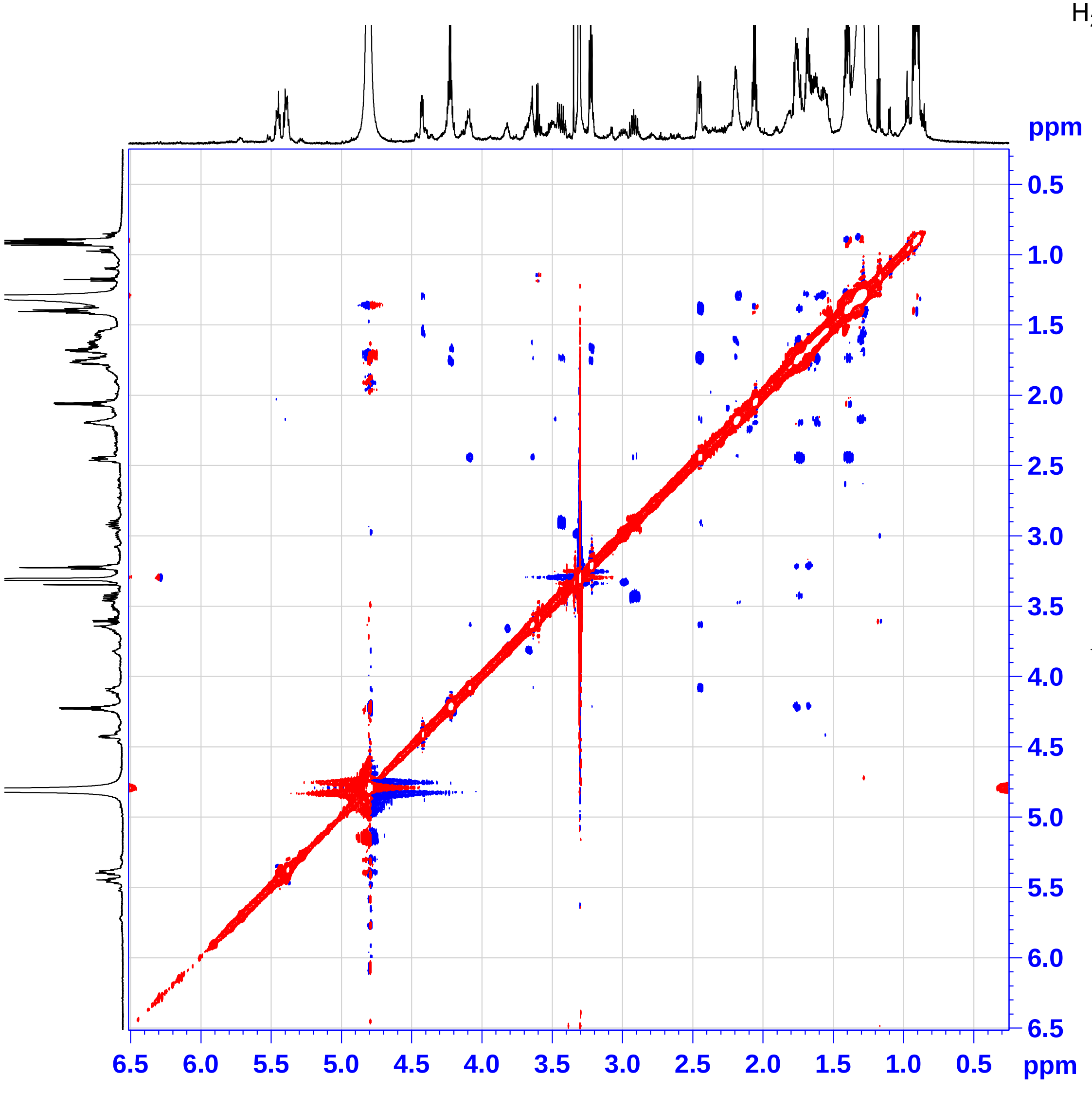

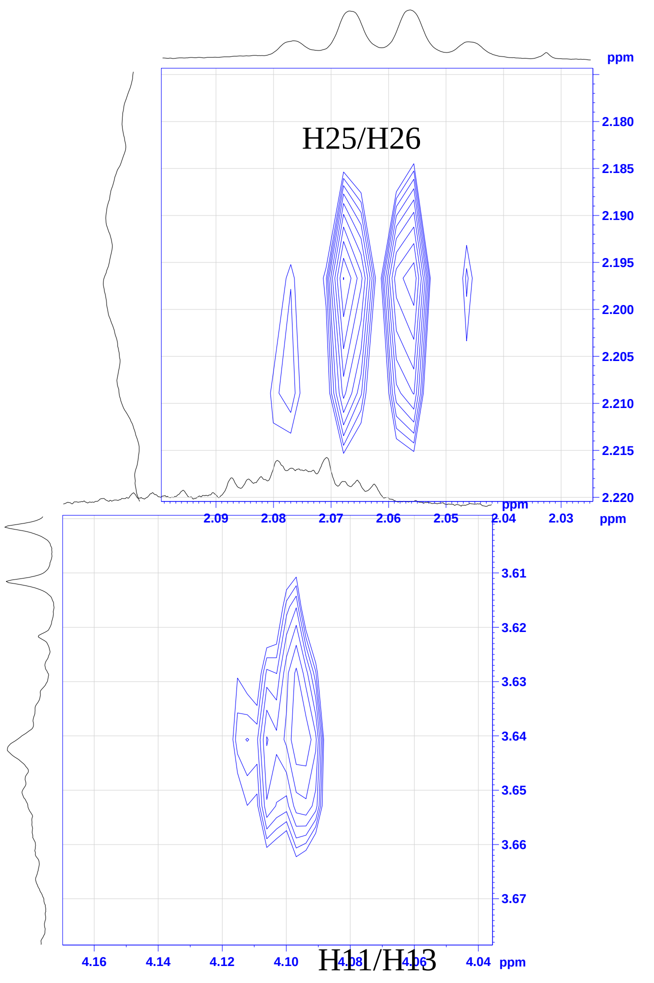

Figure S14. HRESIMS and MS/MS spectra of batzelladine P (**2**) (positive ion mode).

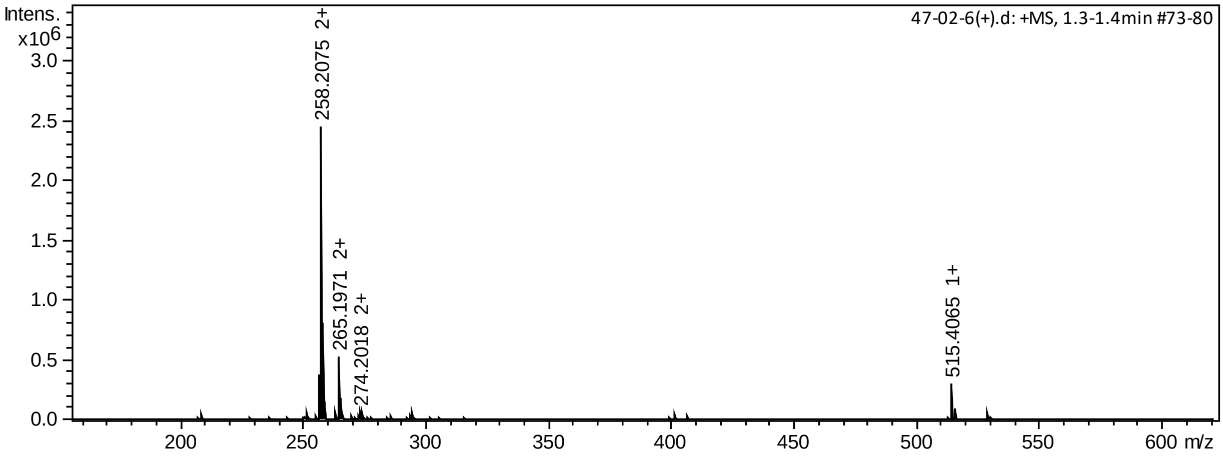

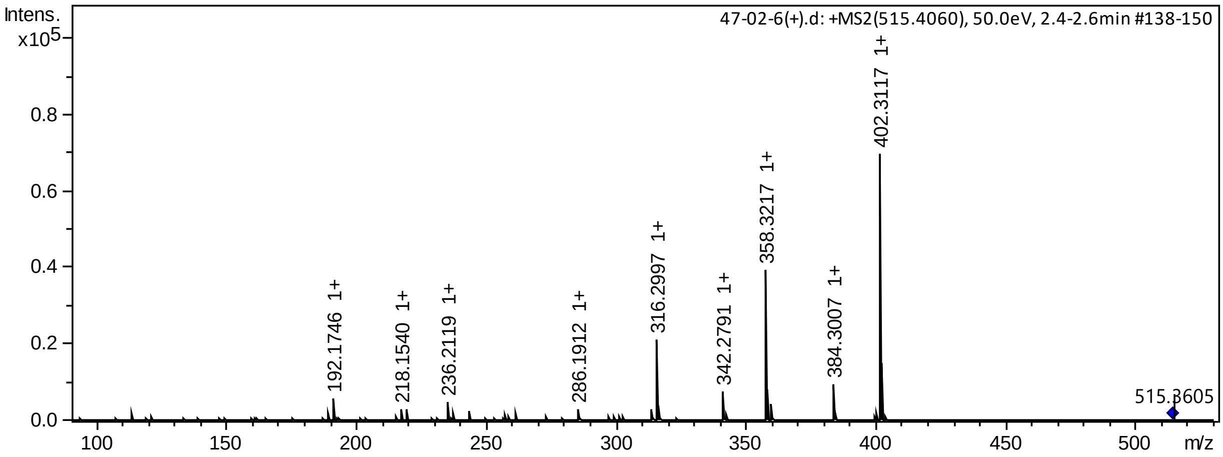

Figure S15. Posible stereoisomers of **1**.

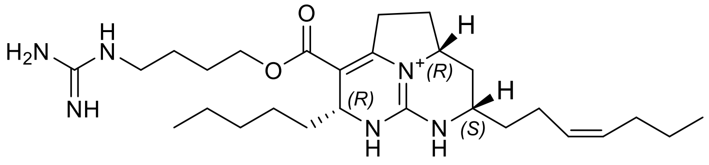

11*R*,13*S*,15*R*-**1a**

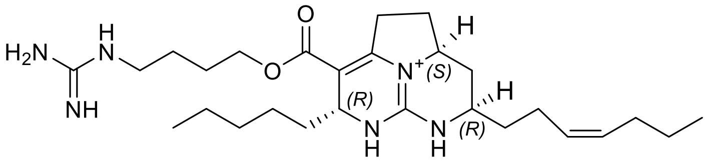

11S,13R,15R-**1b**

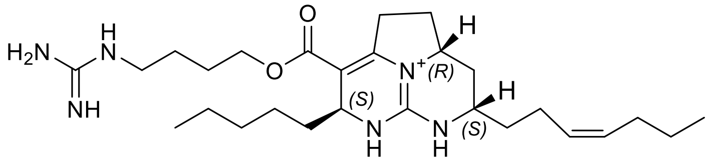

11*R*,13*S*,15*S*-**1c**

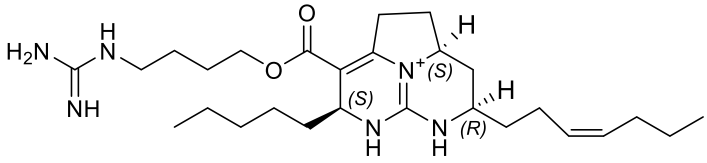

11S,13R,15S-**1d**

Quantum-chemical modeling.

Theoretical modeling of ECD spectra for compound **1** was done using GAISSIAN_16 software [1] as follows. First, the conformational analysis was done and the most stable conformations were selected for stereoisomers 11*R*,13*S*,15*R*-**1a** and 11*S*,13*R*,15*R*-**1b**. This part of investigation was done at B3LYP/6-31G(d)_PCM level of theory with CH_3OH taken as a solvent. The internal rotations of substituents at C(7), C(13) and C(15), as well as the internal rotations of fragments of these substituents around correspondent C–C bonds, and the inversion of ring containing C(13) were accounted for (Scheme 1Q).

Scheme 1Q

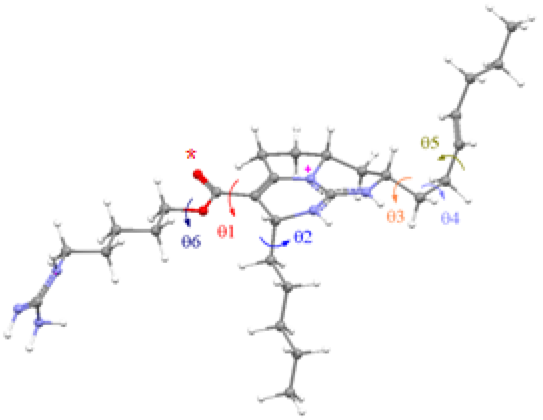

11*R*,13*S*,15*R*-**1a**

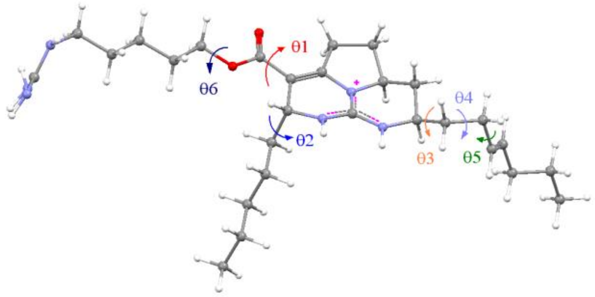

11*S*,13*R*,15*R*-**1b**

Conformations, which electronic energies are in diapason ΔE ≤ 5 kcal/mol, were them chosen for calculation of vertical electronic transitions at TDDFT_ cam-B3LYP/6-311G(d)_PCM//B3LYP/6-31G(d)_PCM level of theory. ECD spectra for each conformation were simulated as a superposition of bands, generated by individual transitions, using GAUSS band-shapes. The used band-width, taken at 1/e of peak height, is $\sigma = 0.34$ eV. The UV shift was taken as $\Delta\lambda = +18$ nm.

The scaled theoretical and experimental ECD spectra were obtained according to equation:

$$\Delta\varepsilon_{scaled}(\lambda) = \Delta\varepsilon(\lambda) \Big/ \left|\Delta\varepsilon(\lambda_{peak})\right| \qquad (1),$$

where the denominator $|\Delta\varepsilon(\lambda_{peak})|$ is a modulo of the peak value for the chosen characteristic band in corresponding ECD spectra ($\lambda_{peak} \approx 250$ nm).

Conformational analysis.

The performed conformational analysis showed, that thermodynamically most stable are conformations with next characteristics:

1. ring containing C(13) has conformation, in which hydrogen atoms are both axial and nearly parallel;
2. the substituents at C(7), C(13) and C(15),predominantly have nearly straight configuration;
3. the substituent at C(15) stay in “trans”- orientation, for which $\theta_2 \equiv \angle C(7)–C(15)–C(16)–C(17) \approx 180°$.

11*S*,13*R*,15*R*-1b and 11*R*,13*S*,15*S*-1c:

The analysis of ECD spectra, calculated for different conformations of stereoisomers of 11*S*,13*R*,15*R*-**1b** and 11*R*,13*S*,15*S*-**1c**, showed that the sign of alone band in the region $235 \le \lambda \le 270$ nm does not change under the influence of conformational rearrangement. This fact allows us to exclude 11*R*,13*S*,15*S*-**1c** stereoisomer from consideration in our further analysis of mutual correspondence between theoretical and experimental ECD spectra (since the sign of band at $\lambda \approx 250$ nm for this stereoisomer is negative). The spectral properties of 11*S*,13*R*,15*R*-**1b** stereoisomer only will be discussed further.

The broad negative band exists in $\lambda \le 235$ nm region in the experimental spectrum. It consists of two (at least) individual bands with peak maximums at $\lambda \approx 210$ and ≈220 nm. The vertical electronic transitions which energies lie in $190 \le \lambda \le 235$ nm region, possess big rotatory strengths. Due to superposition, the bands, generated by individual electronic transitions, are grouped in two separated bands, which may be attributed to two components of negative band in experimental ECD spectrum of **1**. The performed analysis showed that the sign of the high-frequency component of doublet doesn’t change under the influence of conformational rearrangements. On the contrary, the sign of the low-frequency component depends on conformation, in which compound **1** instantaneously exists (manly on the orientation of RO–C=O group at C(7): “*cis*” with $|\theta_1| \approx 0°$ or “*trans*” with $|\theta_1| \approx 180°$; $\theta_1 \equiv \angle O^*–C(6)–C(7)–C(8)$). The intensity of the low-frequency component is comparable with the intensity of the high-frequency component for “*trans*” orientation, but the signs of these two components are opposite each other. For “*cis*” orientation both sub-bands have the same signs, but the intensity of the low-frequency component is for an order (or even more) lower, then the intensity of the high-frequency component. And only for “*cis*”-conformations the correct consequence of band’s signs (negative in $190 \le \lambda \le 235$ nm region and positive for $\lambda \ge 235$ nm)

occurs. These facts denote that the features of ECD spectra, calculated for 11*S*,13*R*,15*R*-**1b** stereoisomer, agree poorly with features of experimental ECD spectrum of **1**.

11*R*,13*S*,15*R*-1a:

The analysis of ECD spectra, calculated for different conformations of 11*R*,13*S*,15*R*-**1a**, showed that conformational rearrangement does not affects the positive sing of alone band in $235 \le \lambda \le 270$ nm region. The sign of band in $270 \le \lambda \le 320$ nm region is more confromationally dependent.

The ratio of intensities for these two bands depends strongly on the orientation of RO–C=O substituent at C(7). For $|\theta_1| \approx 0°$ the low-frequency band at $\lambda \approx 290$ nm is more intensive, then the band at $\lambda \approx 250$ nm. For $|\theta_1| \approx 180°$, on the contrary, the band at $\lambda \approx 250$ nm is more intensive.

In $190 \le \lambda \le 235$ nm region (as it was found earlier for 11*S*,13*R*,15*R*-**1b** stereoisomer), due to superposition, the bands, generated by individual electronic transitions, are grouped in two separated bands, which may be attributed to two components of negative band in experimental ECD spectrum of **1**. The sign of this band for 11*R*,13*S*,15*R*-**1a** after statistical averaging is negative and its intensity is comparable with intensities of positive bands in the $\lambda \ge 235$ region.

REFERENCES

1. Frisch, M.J.; Trucks, G.W.; Schlegel, H.B.; Scuseria, G.E.; Robb, M.A.; Cheeseman, J.R.; Scalmani, G.; Barone, V.; Petersson, G.A.; Nakatsuji, H.; Li, X.; Caricato, M.; Marenich, A.V.; Bloino, J.; Janesko, B.G.; Gomperts, R.; Mennucci, B.; Hratchian, H.P.; Ortiz, J.V.; Izmaylov, A.F.; Sonnenberg, J.L.; Williams-Young, D.; Ding, F.; Lipparini, F.; Egidi, F.; Goings, J.; Peng, B.; Petrone, A.; Henderson, T.; Ranasinghe, D.; Zakrzewski, V.G.; Gao, J.; Rega, N.; Zheng, G.; Liang, W.; Hada, M.; Ehara, M.; Toyota, K.; Fukuda, R.; Hasegawa, J.; Ishida, M.; Nakajima, T.; Honda, Y.; Kitao, O.; Nakai, H.; Vreven, T.; Throssell, K.; Montgomery, J.A.; Jr.; Peralta, J.E.; Ogliaro, F.; Bearpark, M.J.; Heyd, J.J.; Brothers, E.N.; Kudin, K.N.; Staroverov, V.N.; Keith, T.A.; Kobayashi, R.; Normand, J.; Raghavachari, K.; Rendell, A.P. ; Burant, J.C.; Iyengar, S.S.; Tomasi, J.; Cossi, M.; Millam, J.M.; Klene, M.; Adamo, C.; Cammi, R.; Ochterski, J. W.; Martin, R.L.; Morokuma, K.; Farkas, O.; Foresman, J.B.; Fox, D.J. Gaussian 16, Revision A.01; Gaussian, Inc.: Wallingford. CT, USA, **2016**.

Original Western blotting images

Figure 4C

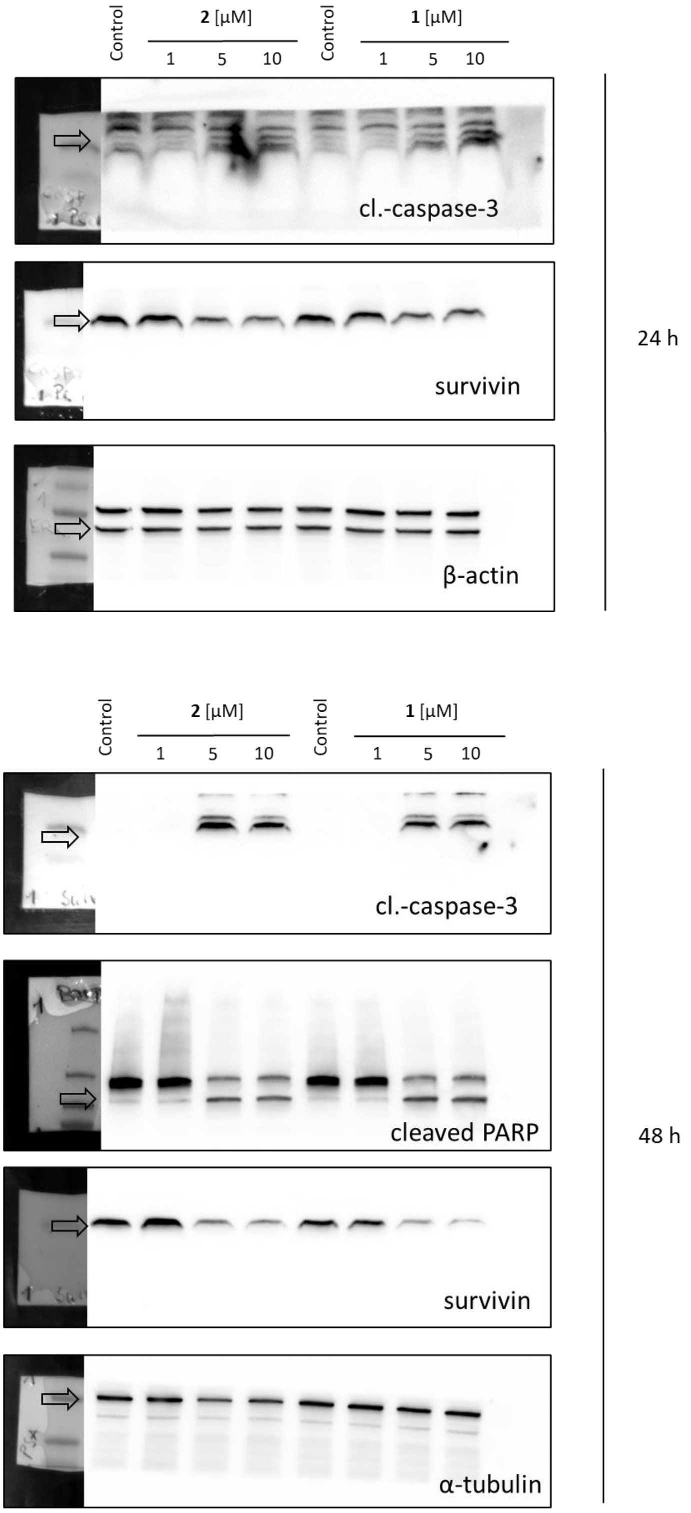

Figure 5A

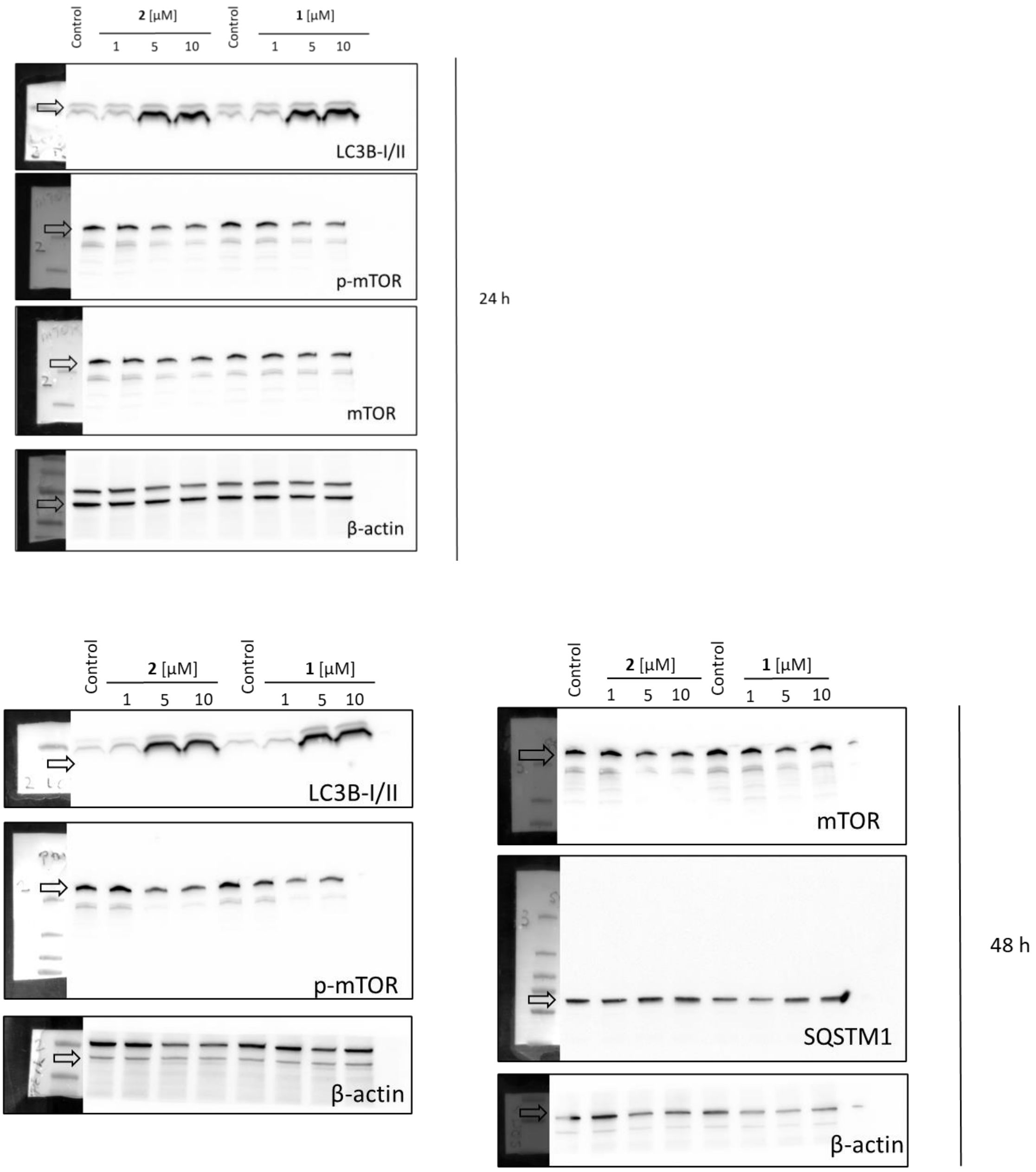
Control
2 [μM]
1 5 10
Control
1 [μM]
1 5 10
LC3B-I/II
p-mTOR
mTOR
β-actin
24 h
Control
2 [μM]
1 5 10
Control
1 [μM]
1 5 10
LC3B-I/II
p-mTOR
β-actin
Control
2 [μM]
1 5 10
Control
1 [μM]
1 5 10
mTOR
SQSTM1
β-actin
48 h
